# Genome-Wide Expression Profiling of Anoxia/Reoxygenation in Rat Cardiomyocytes Uncovers the Role of MitoK_ATP_ in Energy Homeostasis

**DOI:** 10.1155/2015/756576

**Published:** 2015-06-15

**Authors:** Song Cao, Yun Liu, Wenting Sun, Li Zhao, Lin Zhang, Xinkui Liu, Tian Yu

**Affiliations:** ^1^Department of Anesthesiology, Zunyi Medical College, Zunyi 563000, China; ^2^Guizhou Key Laboratory of Anesthesiology and Organ Protection, Zunyi Medical College, Zunyi 563000, China; ^3^Research Center for Medicine & Biology, Zunyi Medical College, Zunyi 563000, China

## Abstract

Mitochondrial ATP-sensitive potassium channel (mitoK_ATP_) is a common end effector of many protective stimuli in myocardial ischemia-reperfusion injury (MIRI). However, the specific molecular mechanism underlying its myocardial protective effect is not well elucidated. We characterized an anoxia/reoxygenation (A/R) model using freshly isolated adult rat cardiomyocytes. MitoK_ATP_ status was interfered with its specific opener diazoxide (DZ) or blocker 5-hydroxydecanote (5-HD). Digital gene expression (DGE) and bioinformatic analysis were deployed. Three energy metabolism related genes (*MT-ND6, Idh2,* and *Acadl*) were upregulated when mitoK_ATP_ opened. In addition, as many as 20 differentially expressed genes (DEGs) were significantly enriched in five energy homeostasis correlated pathways (PPAR, TCA cycle, fatty acid metabolism, and peroxisome). These findings indicated that mitoK_ATP_ opening in MIRI resulted in energy mobilization, which was confirmed by measuring ATP content in cardiomyocytes. These causal outcomes could be a molecular mechanism of myocardial protection of mitoK_ATP_ and suggested that the mitoK_ATP_ opening plays a physiologic role in triggering cardiomyocytes' energy homeostasis during MIRI. Strategies of modulating energy expenditure during myocardial ischemia-reperfusion may be promising approaches to reduce MIRI.

## 1. Introduction

Myocardial infarction has been a leading cause of death worldwide. The prognosis of acute myocardial infarction has been dramatically improved due to the advances of both catheterization techniques and reperfusion therapy by coronary mechanical and pharmacological intervention methods. However, strategies to limit myocardial ischemia-reperfusion injury (MIRI), thus reducing infarct size, have not been well applied in clinical settings.

Although myocardial ischemia-reperfusion (IR) induces lethal injury in the heart, after some artificial interventions, the cardiomyocytes and the heart tissue therein have powerful endogenous mechanisms to protect themselves from oxidative stress, energy deficiency, protein aggregation, and organelle malfunction, thereby minimizing MIRI [1]. For example, Murry et al. in 1990 [2, 3] first proposed that ischemic preconditioning (IPC) may protect the heart by reducing myocardial energy demand during myocardial ischemia and decreasing cell death by preserving ATP content and/or reducing catabolite accumulation. Following the discovery of the mitoK_ATP_ channel locating at the inner mitochondrial membrane in 1991 [4], Garlid et al. and Liu et al. [5, 6] demonstrated it as a trigger of IPC. Pharmacological intervention mimicking the IPC has currently been considered as a promising modality for the treatment of MIRI. Similar myocardial protection can be produced by drugs such as diazoxide (DZ) that open mitoK_ATP_ [5, 7]. Conversely, mitoK_ATP_ blockers (5-hydroxydecanote (5-HD) or glibenclamide) cancelled the effect of preconditioning and pharmacological cardioprotection [5, 6, 8]. It is also demonstrated that the pharmacological inhibition of the mitoK_ATP_ in early reperfusion abolished the infarct-limiting effects of IPost [9–11].

We have reported that mitoK_ATP_ opening was cardioprotective in MIRI [12–14], but our understanding of its specific mechanism remained quite preliminary. To date, the main proposed mechanisms of cardioprotection by mitoK_ATP_ were various: swelling of mitochondria increased fatty acid oxidation (FAO), mitochondrial respiration, and ATP production [15]; inhibition of ATP hydrolysis during ischemia [16, 17] preserved ATP and decreased Ca^2+^ uptake in the cardiomyocytes. However, other endogenous mechanisms of cardioprotection of mitoK_ATP_ activation during IR remain to be elucidated.

Most of the* in vitro* studies used neonatal cardiac cells or immortal cardiac cell lines such as H9c2, which is physiologically different from adult cardiomyocytes [18]. For example, it is reported that neonatal cardiomyocytes were more resistant to hypoxia in comparison to adult ones [19, 20]. So, it may limit the extrapolation of the research results. We developed an A/R model using adult cardiomyocytes freshly isolated from rat to mimic the IR microenvironment* in vivo*; after all, MIRI is present almost exclusively in the adult population.

Compared with microarray and PCR-based technologies, digital gene expression (DGE) platform can provide adequate sequence coverage and quantitative accuracy to capture subtle changes resulting from mitoK_ATP_ opening. In this study, a molecular and bioinformatic pipeline permitted comprehensive analysis of the myocardial mRNA expression. Next-generation sequencing technology was employed and the impact of mitoK_ATP_ on the myocardial transcriptome signature of MIRI was explored to crystallize cardioprotective effects of mitoK_ATP_.

## 2. Materials and Methods

### 2.1. Experimental Animals

Male Sprague-Dawley rats (250–300 g, 16–20 weeks) were provided by the Third Military Medical University (Chongqing, China) and maintained in specific pathogen free (SPF) animal facility in Zunyi Medical College under standardized conditions with 12 h light/dark cycles and free access to rat chow and water. All experimental procedures were performed according to the “Guide for the Care and Use of Laboratory Animals” in China (no. 14924, 2001) and approved by the Experimental Animal Care and Use Committee of Zunyi Medical College.

### 2.2. Isolation of Adult Cardiomyocytes

Rats were anesthetized with sodium pentobarbital (60 mg/kg, combined with 250 U/kg heparin, peritoneal injection). When rats had been successfully anesthetized, the chest cavity was opened and the heart excised rapidly. Ventricular cardiomyocytes were obtained by enzymatic digestion as previously described [24], with some necessary modification. Briefly, hearts were retrogradely perfused with 0.1% type 2 collagenase (Sigma, USA) at constant pressure (9 mL/min/g) on a Langendorff apparatus (Alcott Biotech, China); then the ventricle was scissored out and digested by type 2 collagenase solution in a beaker with manually shaking. Cells were filtered through a piece of gauze and washed 5 times to get rid of collagenase. Cardiomyocytes from one heart were evenly titrated into four 60 mm laminin-precovered Petri dishes. Three mL serum free modified M199 medium (Hyclone, USA, with 2 mM carnitine, 2 mM glutamine, 5 mM taurine, 5 mM creatine, and 0.8 mM EGTA) was added. After 3 hours' incubation, the medium was replaced to get rid of noncardiomyocytes. Cell quality was confirmed with trypan blue exclusion test.

### 2.3. Anoxia/Reoxygenation and DZ Postconditioning in Adult Rat Cardiomyocytes

**Figure 1:**
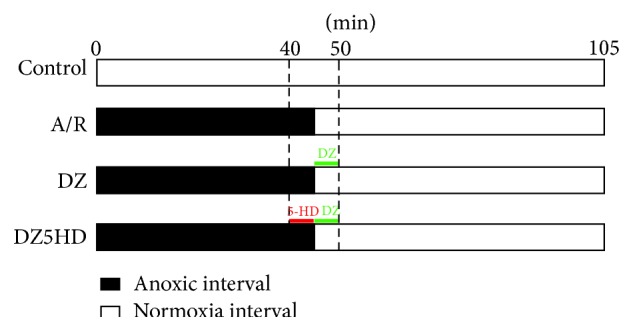
Illustration of the experimental A/R model protocols. Cardiomyocytes were cultured for 20 hours in normoxia incubator. Petri dishes were randomly distributed to 4 groups. Cardiomyocytes of Con were continuously cultured in normoxia environment for 105 min. Medium of A/R group was replaced with N_2_ bubbled (95% N_2_, 5% CO_2_) modified M199 at the 40th min and then replaced with O_2_ bubbled modified M199 at 45th and 50th min. Medium of DZ group was replaced with N_2_ bubbled modified M199 at the 40th min; at 45th min, medium was replaced with O_2_ bubbled modified M199 containing 50 *μ*M DZ and at the 50th min it was replaced with O_2_ bubbled modified M199 to remove DZ. Medium of DZ5HD group was replaced with N_2_ bubbled modified M199 at 40th min containing 100 *μ*M 5-HD; at 45th min, it was replaced with O_2_ bubbled modified M199 containing 50 *μ*M DZ and then replaced with O_2_ bubbled modified M199 to remove DZ at 50th min.

For each test, the 4 Petri dishes were placed in normoxia incubator for 20 hours before being randomly distributed to 4 groups: Control (Con), anoxia/reoxygenation (A/R), diazoxide (DZ), and DZ + blocker 5-hydroxydecanote (5-HD) (DZ5HD). Cardiomyocytes of Con were continuously cultured in normoxia environment for 105 min; A/R: under anoxia for 45 min and then reoxygenated for 60 min; DZ: anoxia for 45 minutes, reoxygenated with 50 *μ*M DZ (Sigma, USA) for 5 min, and then reoxygenated without DZ for another 55 min; DZ5HD: anoxia for 40 min, anoxia with 100 *μ*M 5-HD (Sigma, USA) for 5 min and then reoxygenated with DZ for 5 min and reoxygenated without DZ for another 55 min (Figure 1). Oxygen deprivation and reoxygenation were achieved by series of changes of the medium and incubators. Normoxia was set in a normoxia incubator (O_2_/CO_2_ incubator containing a humidified atmosphere of 5% CO_2_ and 95% air at 37°C). Anoxia was achieved in an anoxic incubator (O_2_/CO_2_ incubator containing a humidified atmosphere of of 5% CO_2_, 1% O_2_, and 94% N_2_ at 37°C) (Figure 1).

### 2.4. Intracellular Free Calcium ([Ca^2+^]_i_) Test

At the end of reoxygenation, [Ca^2+^]_i_ was detected as previously reported [25]. Briefly, M199 was removed; cardiomyocytes of 4 groups were washed twice with PBS and loaded with Fluo-3 AM (Biotium, USA) at a final concentration of 10 *μ*M and incubated for 30 min at 37°C in O_2_/CO_2_ incubator. The solution containing the Ca^2+^ probe was removed and cells were washed twice again with PBS. The average fluorescence intensity of [Ca^2+^]_i_ concentration in labeled cells was detected under a laser scanning confocal microscope (TCS SP2 AOBS, Leica, Germany). The wavelength of excitation was set at 488 nm and the emission wavelength was 525 nm for Fluo-3 fluorescence reading. More than 20 cells from each group were randomly chosen for data analysis; their outlines were circled out and the fluorescence density of Fluo-3 was calculated with Leica confocal software (Leica, Germany).

### 2.5. Cell Viability Detection

Adult cardiomyocytes' viability was detected with Cell Counting Kit-8 (CCK-8, Beyotime, China) in accordance with the manufacturer's instructions. The same amount of cells was seeded into 24-well plates. At the end point of reoxygenation, 30 *μ*L WST-8 solution was added into M199 to form a 3% WST-8 final concentration. Cells were incubated for 1 h before the mixture's OD value was detected at 450 nm wavelength. The replicate size was 6 for each group.

### 2.6. RNA Extraction

At the end of reoxygenation (see Section 2.3), cardiomyocyte samples (3 replicates for 4 groups) were homogenized in TRIzol reagent (Invitrogen, USA) and vortexed with chloroform. The mixture was prepared at room temperature for 2 min and then centrifuged at 12000 ×g at 4°C. The aqueous phase was mixed with 100% ethanol and then filtered with a Qiagen RNeasy column. Subsequent steps for extraction of total RNAs were carried out as the Qiagen RNeasy kit (Qiagen, Germany) instructions described.

### 2.7. Tag Library Construction

The tag-seq library was constructed in accordance with the manufacturer's workflow as previously described [26]. Briefly, 6 mg extracted total RNA was used for mRNA capture with magnetic Oligo (dT) beads. Then cDNA was synthesized and the bead-bound cDNA was subsequently digested with NlaIII. Fragments attached to Oligo (dT) beads were washed away. GEX NlaIII adapter was ligated to the free 5′ end of the digested bead-bound cDNA fragments. Individual cDNA libraries were PCR amplified and purified on a 6% acrylamide gel. Attached DNA fragments were used to create a sequencing flow cell with millions of clusters, which contained about 1000 copies of the templates. Templates were sequenced by the Illumina HiSeq 2500 equipment using the four-color DNA sequencing-by-synthesis (SBS) technology. Each lane generated millions of raw reads.

### 2.8. Data Processing and Statistical Analysis

To obtain high quality and reliable data, raw reads were filtered to remove potentially erroneous reads. Briefly, the 3′ adaptor sequences were trimmed, low-quality tags containing N were abandoned, and small tags and only 1 copy tag were removed before obtaining the clean reads. After filtering, all reads were annotated to Rat Genome V3.4 Assembly (http://rgd.mcw.edu/). All the clean reads were mapped to the reference database; the unambiguous tags were annotated. Copy number of the clean tags of each gene was normalized with the RPKM (reads per kilobase of exon per million mapped reads) method [27] to get the final gene expression.

### 2.9. Identification of DEGs

According to the method by Tarazona et al. [28], the NOISeq-real algorithm was employed to determine the *Q* value (corresponding to the *P* value in differential gene expression detection) and screen genes [29, 30] differentially expressed between Con and A/R, A/R and DZ, and DZ and DZ5HD. In the present study, we considered a gene differentially expressed if the *Q* value was more than 0.8.

### 2.10. Gene Annotation with Gene Ontology and KEGG Pathway

GO (http://www.geneontology.org) provides a dynamic, controlled vocabulary. It comprises 3 independent ontologies: Biological Process, Molecular Function, and Cellular Component, each of which contains hundreds of terms. These terms reflect our understanding of the gene function.

KEGG Pathway database is for systematical analysis of gene functions, linking genomic information with higher order functional information. It provides an indication of the main biochemical and signal transduction pathways that DEGs are involved in.

Finally, the DEGs were enriched with GO (into ontologies and terms) and KEGG Pathway database.

### 2.11. RT-qPCR Analysis

Twenty-five DEGs were randomly selected for real-time quantitative PCR (RT-qPCR). The total RNA used for sequencing was reused to validate DGE sequencing. 500 ng RNA was reverse-transcribed into cDNA using a cDNA synthesis kit (Takara, Japan) in a final volume of 10 *μ*L according to the manufacturer's protocol. RT-qPCR was performed with the CFX Connect Real-Time system (Bio-Rad, USA) using a SYBR green PrimeScript RT kit (Perfect Real Time, Takara, Japan) based on the manufacturer's instructions. The PCR conditions included predenaturing at 95°C for 30 s followed by 40 cycles of denaturation at 95°C for 10 s and combined annealing/extension at 58°C for 30 s. All the mRNA expression levels were calculated based on the comparative quantification method (2^−ΔΔCT^). The *β*-actin gene was used as an internal control. The primer sequences were listed in Table 4.

### 2.12. ATP Quantitation in Cardiomyocytes

At the end of reoxygenation, the cardiomyocytes were scraped off and centrifuged at 1000 ×g for 5 min; the supernatant (M199 medium) was abandoned. 1 mL precooled 0.4 M HClO_4_ was added into the pellet and followed by ultrasonication and centrifugation at 10000 ×g for 20 min. The supernatant was collected and its pH was adjusted to 6.0 to 7.0 with 0.7 mL 1 M K_2_HPO_4_ before centrifugation again at 10000 ×g for another 20 min. All the above-mentioned procedures were conducted at 4°C. The supernatant was filtered through 0.22 *μ*m membrane before high performance liquid chromatography (HPLC) analysis. The chromatographic conditions were as follows: work station: LC 20A (Shimadzu, Japan); column: WondaSil C18-WR (150 mm × 4.6 mm, id = 5 *μ*m; GL Sciences, Japan); column temperature: 25°C; mobile phase: buffered phosphate at pH 7.0; flow rate: 1 mL/min; detection wavelength: 254 nm; sample size: 10 *μ*L. The ATP peaks of samples were determined in reference to the ATP standards (Sigma, USA). The amount of ATP was determined based on the standard curve and regression equation from ATP standard's concentration and peak area. Protein content was measured by using the same sample. ATP level of each sample is normalized to protein content.

### 2.13. Statistical Analysis

The quantitative data were expressed as mean ± SD. For experiments of cardiomyocytes of the four groups, one-way analysis of variance (ANOVA) was performed; LSD or Dunnett's T3 method was used to make multiple comparisons. A *P* value of less than 0.05 was considered to be statistically significant. All data analyses were carried out using SPSS v.19.0 (IBM, USA).

## 3. Results

### 3.1. Isolated Adult Rat Cardiomyocytes

**Figure 2:**
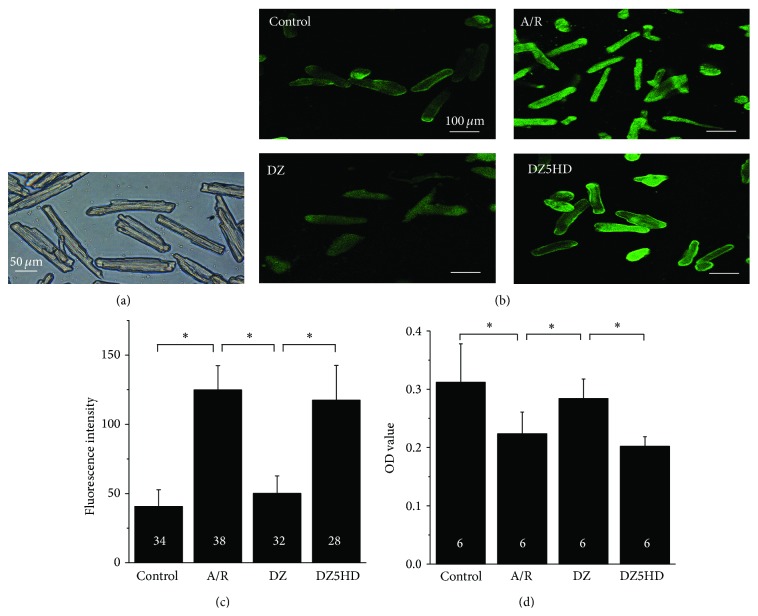
Adult rat cardiomyocytes and their status tests after mitoK_ATP_ opening. (a) Light microscopic morphology of freshly isolated adult cardiomyocytes. They were rod-shaped, with sharp outlines and clear cross striations. (b-c) The effect of DZ and 5HD on the [Ca^2+^]_i_ in adult rat cardiomyocytes. At the end point of reoxygenation, cells of Con, A/R, DZ, and DZ5HD group were pretreated with 10 *μ*M Fluo-3-AM and incubated for 60 min at 37°C and measured with a confocal laser microscope. (b) The [Ca^2+^]_i_ fluorescence image of cardiomyocytes in four groups. (c) The [Ca^2+^]_i_ fluorescence intensity comparison. [Ca^2+^]_i_ in A/R group was increased compared with the Con. Applying of DZ reduced the fluorescence intensity. After 5-HD administration, fluorescence intensity increased. (d) Cell viability test with CCK-8 kit. At the end point of reoxygenation, 30 *μ*L CCK-8 was added into M199 to form a 3% CCK-8 resulting solution. Cells were incubated for 1 h before the mixture's OD value was detected at 450 nm. Cardiomyocytes of A/R group possessed lower level of cell viability when compared with Con. DZ group contained higher level of cell viability when compared with A/R group. Cells in DZ5HD showed the lowest level of cell viability in the 4 groups. Data are mean ± SD. Replication number for each group is marked on the columns. ^*^ *P* < 0.05.

A high percentage (70–80%) of rod-shaped adult cardiomyocytes with clear striations and sharp outlines without visible vesicles were obtained with our method (Figure 2(a)).

### 3.2. [Ca^2+^]_i_ and Cell Viability Detection

We used Fluo-3 AM to examine Ca^2+^ mobilizations in cardiomyocytes. In Con group, the level of [Ca^2+^]_i_ was the lowest. Compared with Con, [Ca^2+^]_i_ increased significantly in A/R (*P* < 0.05). After the applying of DZ, [Ca^2+^]_i_ fluorescence decreased dramatically (*P* < 0.05) compared with A/R while there was an apparent increase (*P* < 0.05) in DZ5HD compared with DZ (Figures 2(b) and 2(c)). It indicated that DZ strongly inhibited the [Ca^2+^]_i_ levels in adult rat cardiomyocytes.

Cardiomyocytes in A/R group possessed lower level of cell viability (*P* < 0.05) when compared with Con. DZ group contained higher level of cell viability (*P* < 0.05) when compared with A/R group, while DZ5HD group showed lower level of cell viability (*P* < 0.05) when compared with DZ group (Figure 2(d)).

### 3.3. Quality Evaluation of DGE Reads

A summary of the DGE reads and their mapping to the rat genome database is presented in Supplementary Table 1  available online at  http://dx.doi.org/10.1155/2014/756576. For each group, more than 4.4 million clean reads were sequenced. Low-quality reads accounted for no more than 1.6% and modified Q30 bases rate no less than 97% in all the 12 libraries (Supplementary Table 1). Besides, perfect matched reads accounted for 60% and unique matched reads occupied more than 70% of all reads mapping to rat genome (Table 1), which revealed that the sample preparation and the sequencings were in perfect condition.

### 3.4. Sequencing Saturation Analysis

Samples with replicates of sequencing, sequencing saturation analysis can be performed to test whether the detected genes' percent increased with total reads number. As shown in Supplementary Figure 1, for 3 replicates of 4 groups, when the total tag number came to 3 million, the genes number started to level out. When the total tag number reached 4 million, gene number inclined to stabilization. It suggested that no more distinct genes would be identified when the total clean reads reached a certain number. For all of the 12 libraries, there were more than 4.4 million clean reads (Supplementary Table 1), which indicated that the deep sequencing results were comprehensive and saturated.

### 3.5. DEGs between Groups

All genes annotated to the rat genome (Supplementary Excel 1) were analyzed for an evidence of differential expression. A detailed description of DEGs between two groups was presented in Supplementary Excel 2 (Con versus A/R), Excel 3 (A/R versus DZ), and Excel 4 (DZ versus DZ5HD). Those genes were to some extent differentially expressed; they were considered significant with a *Q* value more than 0.8. A list of the top 10 DEGs between two groups was shown in Table 2. In these genes,* Mt-nd6*,* Acadl,* and* Idh2* are energy metabolism correlated and their expression status is listed in Table 3.

### 3.6. RT-qPCR Analysis

**Figure 3:**
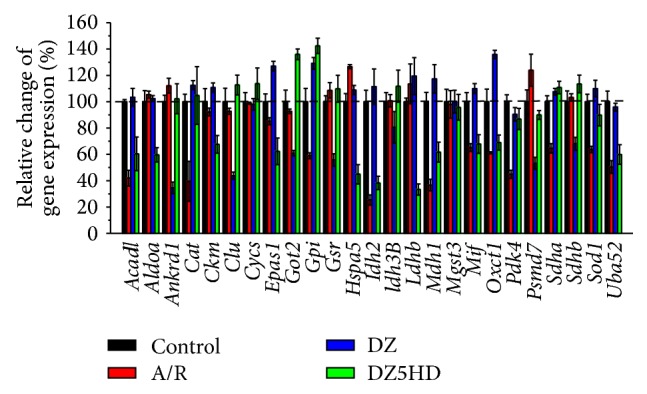
Validating DGE outcomes with RT-qPCR. 25 genes were randomly selected and RT-qPCR tests were carried out. Except* Cycs, Idh3B, Mgst3,* and* Pdk4*, all of the genes' expression was well in accordance with the results from Illumina sequencing (Table 4). Data are mean ± SD. All experiments were done in triplicate.

To confirm the DEGs revealed by the Illumina sequencing, 25 genes were randomly selected (Table 4) and assayed by SYBR green based RT-qPCR (Figure 3). Except* Cycs, Idh3B, Mgst3,* and* Pdk4*, 21 out of the 25 genes were expressed well in accordance with the results from Illumina sequencing (Table 4).

### 3.7. GO Enrichment Analysis

**Figure 4:**
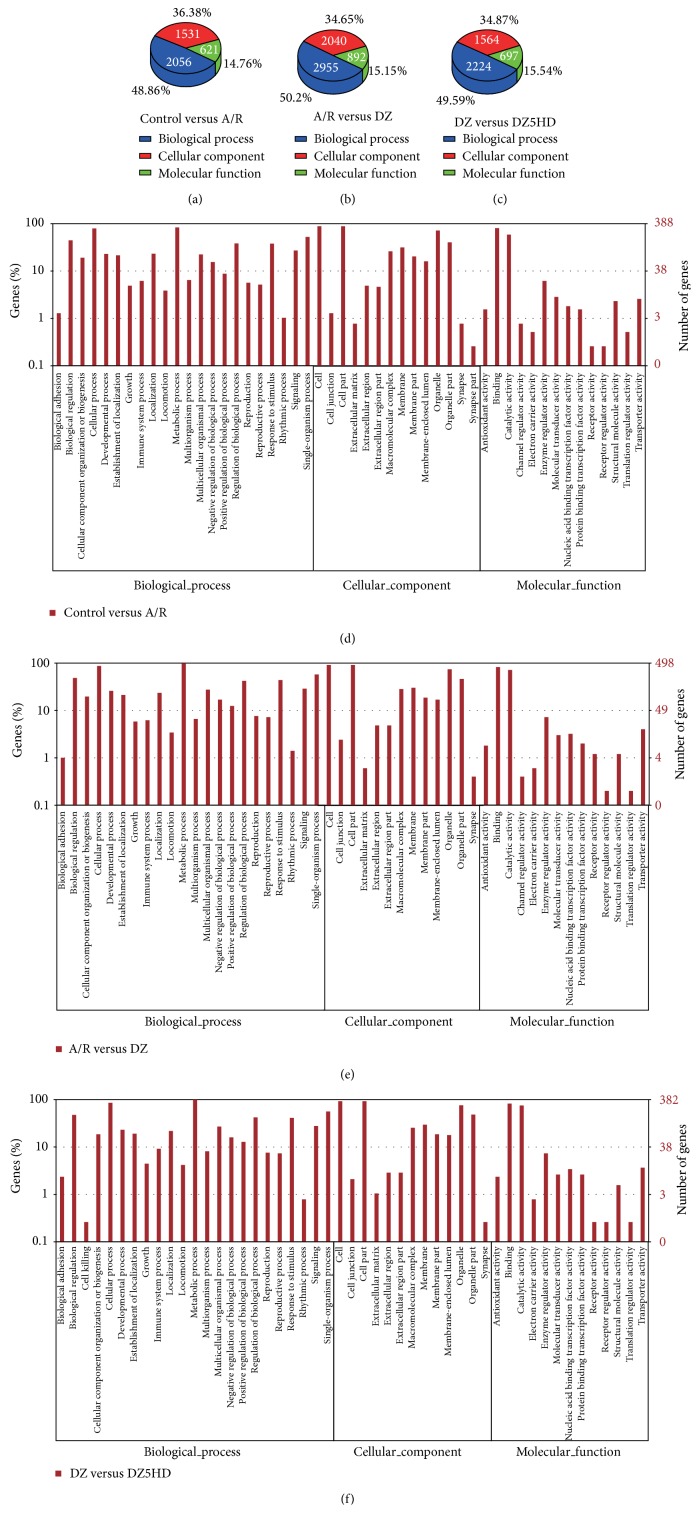
Ontology and term enrichment of DEGs in Gene Ontology. (a–c): Ontology enrichment for Con versus A/R, A/R versus DZ, and DZ versus DZ5HD. Most of the DEGs from Con versus A/R (2056 DEGs, 48.86%), A/R versus DZ (2955, 50.2%), and DZ versus DZ5HD (2224, 49.59%) participated in the Biological Process ontology. (d–f): Histogram presentation of Gene Ontology functional classification and DEGs' enrichment ((d) Con versus A/R; (e) A/R versus DZ; (f) DZ versus DZ5HD). The results are summarized in three main categories: Biological Process, Cellular Component, and Molecular Function. The *y*-axis on the right is the number of DEGs in a category. The *y*-axis on the left is the percentage of a specific category of genes in the main category. For significantly enriched terms (Con versus A/R, A/R versus DZ, and DZ versus DZ5HD), see Supplementary Tables 2–4.

Ontology and term enrichment of DEGs in GO is listed in Figure 4. GO enrichment showed many of the DEGs from Con versus A/R (Figure 4(a)), A/R versus DZ (Figure 4(b)), and DZ versus DZ5HD (Figure 4(c)) participating in the Biological Process ontology. Histogram presentation of Gene Ontology functional classification and DEGs' enrichment is shown in Figures 4(d)–4(f) ((d) Con versus A/R; (e) A/R versus DZ; (f) DZ versus DZ5HD). The significantly enriched (corrected *P* < 0.05) terms for Con versus A/R (Supplementary Table 2), A/R versus DZ (Supplementary Table 3), and DZ versus DZ5HD (Supplementary Table 4) also listed.

### 3.8. Pathway Analysis

KEGG Pathway provides an indication of the main biochemical and signal transduction pathways that DEGs are involved in. Pathway enrichment for Con versus A/R, A/R versus DZ, and DZ versus DZ5HD were displayed in Supplementary Tables 5–7. For Con versus A/R, A/R versus DZ, and DZ versus DZ5HD, there were 40, 48, and 37 pathways highly enriched (*P* < 0.01, *Q* < 0.05), respectively.

In all the pathways, Metabolic Process was the DEGs most enriched one. It is not difficult to notice that many energy metabolism correlated pathways, such as fatty acid metabolism pathway, TCA cycle, proteasome, PPAR signaling pathway, and peroxisome pathway were highly enriched (Table 5).

### 3.9. ATP Detection

**Figure 5:**
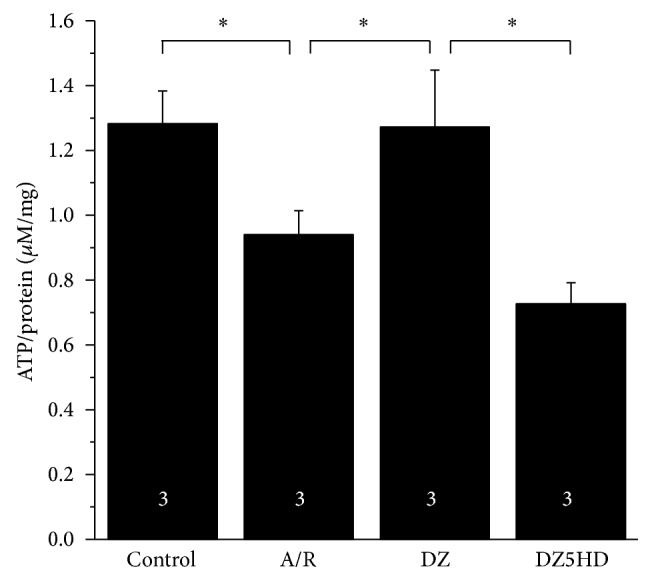
ATP quantitation. At the end of reoxygenation, ATP content was detected by HPLC at a detection wavelength of 254 nm. Protein content was also measured by using the same sample. ATP level of each sample was normalized to protein content of the same sample. Data are mean ± SD. *n* = 3 for each group. ^*^ *P* < 0.05.

At the end of reoxygenation, the ATP levels of the A/R groups were much lower than Con (*P* < 0.05); ATP concentrations of DZ groups were much higher than A/R groups (*P* < 0.05). When 5-HD was applied, the beneficial effect of DZ was abolished in DZ5HD when compared with DZ (*P* < 0.05) (Figure 5).

## 4. Discussion

MIRI is always one of the leading causes of morbidity.* In vitro* experimental A/R model is a powerful tool to mimic* in vivo* ischemia-reperfusion injury. We developed an A/R model using freshly isolated adult rat cardiomyocytes, which are more relevant to the* in vivo* IR conditions.

In present study, DGE and bioinformatics technologies were employed to analyse molecular change after A/R and mitoK_ATP_ opening. Our results demonstrated not only the robustness of next-generation sequencing in exploring the molecular change resulting from mitoK_ATP_ opening but also the potential of the combine of next-generation sequencing and KEGG Pathway analysis to provide clues into target finding of molecular mechanisms underlying the myocardium protective effect of mitoK_ATP_.

MitoK_ATP_ opening or closing in cultured adult rat cardiomyocytes significantly resulted in gene expression change. Many of the genes were energy related. Metabolic Process was the DEGs most enriched GO ontology and energy metabolism correlated pathways were highly enriched too. We could not help doubting that mitoK_ATP_ might have interfered with the energy metabolism and we confirmed that by directly measuring ATP content of four groups at the end of reoxygenation.

Three energy metabolism correlated genes,* Mt-nd6*,* Idh2,* and* Acadl,* were all upregulated (A/R versus DZ).* Mt-nd6* encodes NADH-quinone oxidoreductase (complex I) subunit 6 in mammal. In the respiratory chain, complex I is responsible for the oxidation of NADH and contributes to the formation of the proton gradient which drives ATP synthesis and passes electrons to ubiquinone [31]. Ischemia-reperfusion injury was characterized by decreased complex I respiration [32]. In this study, expression of* Mt-nd6* decreased after A/R treatment, while it was upregulated tremendously in DZ compared with A/R. Complex I is extremely susceptible to oxidative damage and subsequently produces more ROS [33], leading to extensive mitochondrial dysfunction and the depletion of ATP. MitoK_ATP_ opening by DZ increased* Mt-nd6* expression, which might have contributed to ATP synthesis and resulted in its myocardial protection.

*Idh2* encodes isocitrate dehydrogenase in mitochondria. In present study, expression of* Idh2* varyed: Con versus A/R downregulated; A/R versus DZ upregulated; DZ versus DZ5HD downregulated. Isocitrate dehydrogenase is the rate-limiting enzyme of TCA cycle, which catalyzes the oxidative decarboxylation of isocitrate to 2-oxoglutarate. Isocitrate dehydrogenase plays a role in intermediary metabolism and energy production. It had been reported that isocitrate dehydrogenase activity increased at the ischemia region when heart underwent ischemia [34, 35]; the authors deemed the increase that came from the increased need of energy.

Long Chain Acyl-CoA Dehydrogenase (*Acadl*) encodes long chain acyl-CoA dehydrogenase, which catalyzes the *α*- and *β*-dehydrogenation of acyl-CoA esters in fatty acid metabolism. It is the first rate-limiting enzyme in fatty acid *β*-oxidation reaction [36]. In present study,* Acadl* was downregulated after A/R, upregulated when mitoK_ATP_ opened, and downregulated again when mitoK_ATP_ was blocked by 5-HD. In physiological state, 60–70% of the total energy the heart needs comes from fatty acid *β*-oxidation [37]. In the ischemic condition, FAO is more indispensable. Ito et al. [38] demonstrated that high levels of fatty acids in the perfusate were capable of enhancing postischemic energy production and increasing contractile function. That study provided evidence that, in heart with limited oxidative capacity, increasing exogenous energy substrate supply and boosting FAO generated more ATP and quickly normalized energy production. From what is mentioned above, mitoK_ATP_ opening may alleviate the energy depletion when adult cardiomyocytes underwent A/R injury by boosting the fatty acid *β*-oxidation.

6 energy correlated pathways, Peroxisome pathway, PPAR signaling pathway, citrate cycle (TCA cycle) pathway, fatty acid metabolism pathway, and proteasome pathway were DEGs significantly enriched (*P* < 0.01).

TCA cycle and fatty acid metabolism directly generate energy. 7 DEGs from Con versus A/R were enriched in TCA cycle pathway. They were all downregulated after A/R injury. When mitoK_ATP_ was open, all of them were upregulated. It is obvious that A/R suppressed TCA cycle. This could be one of the reasons why A/R decreased ATP content. We could see that DZ saved TCA cycle. 8 DEGs from Con versus A/R and 14 DEGs from A/R versus DZ, including* Acadl*, were enriched in fatty acid metabolism pathway. It seemed that A/R suppressed these two pathways and mitoK_ATP_ reinforced them.

Peroxisome proliferator-activated receptors (PPARs), especially PPAR-*α*, are sensitive to fatty acids and their derivatives. They are also ligand-activated transcription factors regulating cardiac FAO and energy homeostasis [39, 40]. PPAR-*α* is expressed highly in the heart and evidence had showed that PPAR-*α* was involved in the regulation of numerous genes encoding FAO enzymes [41]. Overexpression of PPAR-*α* and its target metabolic genes promoted FAO as a source of energy under conditions of acute IR [42, 43]. Besides its well-known action on cardiac energy metabolism and lipid homeostasis, emerging evidence indicated that administration of PPAR-*α* synthetic ligands was myocardial protective in an IR setting, as manifested by improved postischemic recovery of contractile function and reduced infarct size in both* in vivo* and* ex vivo* models [42, 44]. Mice overexpressing PPAR-*α* in heart displayed increased FAO rates, accumulated triacylglycerides, and decreased glucose metabolism, and they eventually developed cardiomyopathy [45, 46]. Not surprisingly, mice lacking PPAR-*α* had elevated free fatty acid levels as a consequence of inadequate FAO, rendering them hypoglycemic as a result of their reliance on glucose [47]. In present study, although PPAR-*α* gene did not change after mitoK_ATP_ opening, 17 DEGs from A/R versus DZ were enriched in peroxisome pathway (*P* = 1.3 × 10^−7^) and 16 DEGs significantly enriched in PPAR signaling pathway (*P* = 9.9 × 10^−7^). 12 DEGs from DZ versus DZ5HD were enriched in (*P* = 1.1 × 10^−6^) peroxisome pathway and 14 enriched in PPAR signaling pathway (*P* = 2.4 × 10^−5^). Nevertheless, to test the relative activity change of PPAR-*α* after mitoK_ATP_ opening, further studies are needed.

The ubiquitin proteasome system (UPS) degrades targeted abnormal and most normal proteins in cells. Most degradation via the UPS is ATP-dependent. This process involves ubiquitin ligases E1, E2, and E3, which function in concert with chaperones to identify and ubiquitinate appropriate target proteins [48–50]. Then the resulting polyubiquitinated proteins are transferred to the 26S proteasome, where they are degraded into peptides and ubiquitin. Proteasome pathway enriched 14 DEGs from Con versus A/R; 13 DEGs were downregulated in A/R group while 7 DEGs upregulated after mitoK_ATP_ opening. It is obvious that A/R induced the downregulation of UPS and mitoK_ATP_ opening reactivated it. Proteasome that functioned insufficiently had been observed most consistently in MIRI [51, 52]. Such studies supported the hypothesis that IR decreased proteasome activity by reducing ATP levels, as well as oxidative unfolding and damaging proteasome proteins [53]. To test this hypothesis, proteasome gain-of-function or loss-of-function studies in animal models of MIRI were carried out. However, the results showed a paradox: gain-of-function using transgenic mice with increased proteasome activity showed protection from MIRI [54], whereas loss-of-function studies using pharmacological means also revealed protection from MIRI [55–57]. In present study, 13 DEGs were downregulated after A/R (Con versus A/R); this should be a feedback of ATP depletion resulting from A/R. 7 DGEs were upregulated in DZ group (A/R versus DZ); this could be a consequence of ATP recovery after mitoK_ATP_ opening.

**Figure 6:**
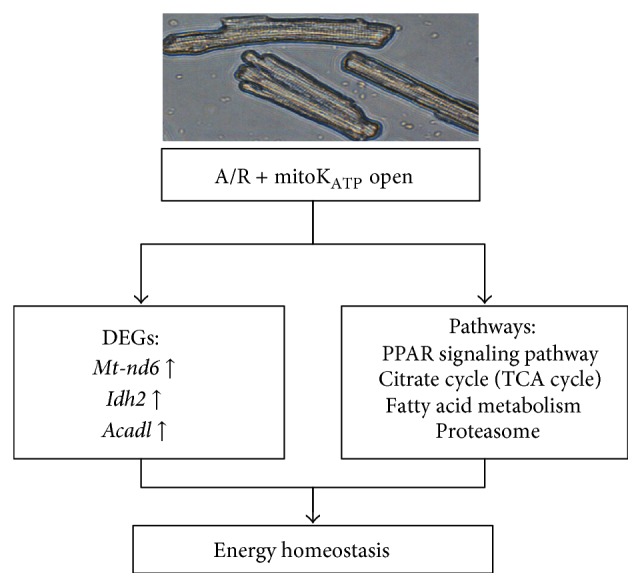
Scheme of the molecular myocardial protective mechanism of mitoK_ATP_ in myocardial A/R setting. MitoK_ATP_ opening-up regulated the expression of* Mt-nd6*,* Idh2,* and* Acadl*. In addition, mitoK_ATP_ opening may recruit DEGs to regulate PPAR signaling pathway, TCA, fatty acid metabolism, and proteasome pathways. Eventually, mitoK_ATP_ opening resulted in an energy homeostasis in the adult rat A/R cardiomyocytes.

Energy was so desperately needed in A/R environment that Metabolic Process was the most enriched GO ontology in Con versus A/R, A/R versus DZ, and DZ versus DZ5HD. In addition, energy metabolism related genes and pathways were significantly interfered with each other (Figure 6). UPS is protein related; TCA cycle pathway controls aerobic metabolism of glucose, PPAR-*α*, and Acadl effect on *β*-dehydrogenation of acyl-CoA esters in FAO. To sum up, mitoK_ATP_ regulated the metabolism of 3 main nutriments: glucose, fatty acid, and protein and kept a balance between energy production and consumption at the setting of A/R in adult cardiomyocytes. Strategies to increase energy supply in MIRI may be a good choice. Metabolism correlated genes and pathway nodes may be promising therapeutic targets. At the same time, we must confess that, to assure the effects of specific gene and signaling pathway mentioned above in MIRI, further gain- or/and loss-of-function studies will be needed.

## Supplementary Material

Excel 1: Raw data of the expression of all the gene (4 groups × 3 replicates of the sequencing) .
Excel 2: Significant differently expressed genes from Control versus A/R.
Excel 3: Significant differently expressed genes from A/R versus DZ.
Excel 4: Significant differently expressed genes from DZ versus DZ5HD.
Supplementary Figure 1: Assessment of the DGE saturation degree.
Supplementary Table 1: The raw data of DGE profile of 12 sequencings.
Supplementary Table 2: DEGs significantly enriched GO terms from Control vs A/R (P<0.05).
Supplementary Table 3: DEGs significantly enriched GO terms from A/R vs DZ (P<0.05).
Supplementary Table 4: DEGs significantly enriched GO terms from DZ vs DZ5HD (P<0.05).
Supplementary Table 5: Significantly enriched pathways for DEGs from Con vs A/R (P<0.01).
Supplementary Table 6: Significantly enriched pathways for DEGs from A/R vs DZ (P<0.01).
Supplementary Table 7: Significantly enriched pathways for DEGs from DZ vs DZ5HD (P<0.01).

## Figures and Tables

**Table 1:** Summary of DGE profile and reads' mapping to the rat genome.

Library number	Total reads (%)	Total base pair (%)	Total mapped reads (%)	Perfect match (%)	≤2 bp mismatch (%)	Unique match (%)	Multiposition match (%)	Total unmapped reads (%)
Con-1	4350000, 100%	213120000, 100%	3690000, 84.92%	2690000, 61.92%	1000000, 23.00%	3470000, 79.81%	220000, 5.10%	650000, 15.08%
Con-2	5180000, 100%	253940000, 100%	4130000, 79.86%	3110000, 60.19%	1010000, 19.66%	3750000, 72.47%	380000, 7.38%	1040000, 20.14%
Con-3	4760000, 100%	233230000, 100%	3890000, 81.74%	2840000, 59.76%	1040000, 21.98%	3550000, 74.67%	330000, 7.07%	860000, 18.26%
A/R-1	5310000, 100%	260380000, 100%	4280000, 80.55%	3250000, 61.34%	1020000, 19.21%	3890000, 73.21%	390000, 7.34%	1030000, 19.45%
A/R-2	4600000, 100%	225450000, 100%	3760000, 81.85%	2800000, 61.07%	950000, 20.78%	3440000, 74.81%	320000, 7.03%	830000, 18.15%
A/R-3	5040000, 100%	246860000, 100%	4180000, 83.12%	3050000, 60.64%	1130000, 22.48%	3810000, 75.66%	370000, 7.46%	850000, 16.88%
DZ-1	5110000, 100%	250260000, 100%	4200000, 82.42%	3180000, 62.28%	1020000, 20.15%	3860000, 75.71%	340000, 6.72%	890000, 17.58%
DZ-2	5010000, 100%	245250000, 100%	4080000, 81.52%	3050000, 60.94%	1030000, 20.58%	3720000, 74.32%	360000, 7.20%	920000, 18.48%
DZ-3	4900000, 100%	240220000, 100%	4060000, 82.84%	2980000, 60.93%	1070000, 21.91%	3680000, 75.09%	380000, 7.76%	840000, 17.16%
DZ5HD-1	4900000, 100%	240100000, 100%	3940000, 80.58%	2960000, 60.58%	980000, 20.01%	3620000, 73.89%	320000, 6.69%	950000, 19.42%
DZ5HD-2	4720000, 100%	231050000, 100%	3770000, 80.12%	2860000, 60.80%	910000, 19.32%	3390000, 71.96%	380000, 8.16%	930000, 19.88%
DZ5HD-3	4470000, 100%	218750000, 100%	3670000, 82.37%	2680000, 60.21%	980000, 22.16%	3350000, 75.16%	320000, 7.21%	780000, 17.63%

**Table 2:** Top 10 DEGs from Con versus A/R, A/R versus DZ, and DZ versus DZ5HD.

Number	Con versus A/R			A/R versus DZ			DZ versus DZ5HD		
	Gene name	log_2_^Ratio (A/R/Con)^⁡	*Q* value	Gene name	log_2_^Ratio (DZ/A/R)^⁡	*Q* value	Gene name	log_2_^Ratio (DZ5HD/DZ)^⁡	*Q* value
1	Pdlim2	4.95	0.84	Idh2	5.02	0.95	Ivd	4.22	0.92
2	MT-ND6	−4.94	0.94	Oxct1	4.92	0.95	MT-ND6	−3.95	0.91
3	Aldh1a7	−4.92	0.82	Acadl	4.87	0.94	Atf4	3.92	0.91
4	Idh2	−4.83	0.94	Mdh1	4.84	0.94	Ldhb	−3.85	0.91
5	Uba52	−4.71	0.93	Mdh2	4.73	0.94	Clu	3.84	0.90
6	Mdh2	−4.68	0.93	Aldh16a1	4.67	0.86	Idh2	−3.75	0.90
7	Podnl1	−4.65	0.80	Podnl1	4.61	0.83	Ankrd1	3.74	0.90
8	Mdh1	−4.63	0.93	Omg	4.60	0.82	Podnl1	−3.73	0.80
9	RGD1311224	−4.59	0.81	MT-ND6	4.56	0.93	Acadl	−3.69	0.90
10	Acadl	−4.58	0.93	Uba52	4.56	0.93	RGD1311224	−3.66	0.81

**Table 3:** Three energy metabolism related DEGs.

Gene name	Gene description	Con versus A/R		A/R versus DZ		DZ versus DZ5HD	
		log_2_^Ratio (A/R/Con)^⁡	*Q* value	log_2_^Ratio (DZ/A/R)^⁡	*Q* value	log_2_^Ratio (DZ5HD/DZ)^⁡	*Q* value
MT-ND6	NADH dehydrogenase subunit 6 (mitochondrion)	−4.94	0.94	4.56	0.93	−3.95	0.91
Idh2	Isocitrate dehydrogenase 2 (NADP+), mitochondrial	−4.83	0.94	5.02	0.95	−3.75	0.90
Acadl	Acyl-CoA dehydrogenase, long chain	−4.58	0.93	4.87	0.94	−3.69	0.90

**Table 4:** Genes selected for RT-qPCR confirmation.

Gene name	Primer sequences (5′ to 3′)		Reads number (RPKM, mean from 3 sequencings)			
	Forward	Reverse	Con	A/R	DZ	DZ5HD
Acadl	GGAATGAAAGCCCAGGACACAG	TCAAACATGAACTCACAGGCAGAAA	421.61	17.57	515.82	39.71
Aldoa	GGTGGTGTTGTGGGCATTAAGGT	ATGGCGAGGGACGAGGGAGTA	1171.38	1358.29	1300.62	121.90
Ankrd1	AAAATCAGTGCCCGAGACAAGC	ACCGAAGGTCATCAAGAGCCG	2875.32	3262.76	215.95	2890.14
Cat	GGCACACTTTGACAGAGAGCGG	CTGTGGAGAATCGGACGGCA	143.64	11.32	149.61	152.13
Ckm	AACCCACAGACAAGCATAAGACC	CTTCCACGGACAGCTTCTCTACA	862.02	929.51	903.35	139.07
Clu	ACTCAGAAGTCCCCTCTCGTGT	TTTCCTGCGGTATTCCTGTAGC	1009.00	1068.16	73.26	1050.66
Cycs	AAGCATAAGACTGGACCAAACCTC	GTGATACCTTTGTTCTTGTTGGCAT	230.49	289.74	129.06	283.52
Epas1	ACCTTCCCAGCCACCATCTACC	ACTTGCCACTCCTGACCCCTTT	10.16	10.53	10.68	1.89
Got2	GGGACTGGCTGATTTTTGTAAGG	CAGAAAGACATCTCGGCTGAACT	241.93	277.84	54.72	275.22
Gpi	ACCCAGGAGACCATCACCAAC	CTACCCAATCCCAGAACTCGAAC	137.57	10.77	146.16	141.82
Gsr	GTTGTGTTTTTCTTGCTTTGGC	GGAGGATTCTGAGTTGTTTGAGG	15.80	21.76	4.91	22.58
Hspa5	ACACTTGGTATTGAAACTGTGGGAG	CTTGATTGTTACGGTGGGCTG	132.59	171.78	157.12	27.08
Idh2	CCCATCACCATTGGCAGACAC	CCTCCGGCAGGGAAGTTATACA	718.92	25.15	816.33	60.47
Idh3B	ATTCGAGAACAGACAGAAGGGGAGT	CTCTGAGACTTGGTTCGAGTGACG	170.23	210.25	28.79	205.45
Ldhb	ACCAGAAGCTGAAGGACGATGAG	TGACCTACGTACAAGGCCGAAGA	1337.06	1599.30	1535.85	106.07
Mdh1	TCTCCTCCGCATGACTACACAG	TAGATCGCAGCACTAACAACGT	445.93	17.92	513.76	130.68
Mgst3	AAAGCCCGCAAGAAGTACAAGGT	CACGGTTAGGAAGAATAGGAAGGG	175.29	183.50	178.22	75.55
Mif	TATTACGACATGAACGCAGCCAA	TCAAACCATTTATTTCTCCCGACC	75.60	19.21	82.05	22.40
Oxct1	ATTGTAGACATTGGCTCGTTTGCTC	TTGGCTTTTCCTTCACCTTCCTTT	224.68	10.00	303.23	43.54
Pdk4	CAAGTCAGCCTTCAAACATTATCA	AAACAAGAGTCCACACACATTCA	184.97	14.36	193.38	32.08
Psmd7	AAGAGCGATGCGAAGAAAGAGGA	AAGGGTGACCAGGGCAGAGAG	74.87	82.47	13.07	59.27
Sdha	CTCTTTCCTACCCGCTCACATAC	TGTCATAGAAATGCCATCTCCAG	209.87	17.69	263.44	254.47
Sdhb	TCAACGGAGGCAACACGCT	GCATAGAAGTTACTCAAGTCAGGGA	378.70	425.40	36.17	419.09
Sod1	GGCTTCTGTCGTCTCCTTGCTT	CTGGTTCACCGCTTGCCTTCT	186.25	21.37	199.90	198.85
Uba52	ACCCTGTCCGACTACAACATCCA	TGTACTTCTGGGCAAGCTGACGA	618.42	23.57	556.13	51.39

**Table 5:** DEGs highly enriched and energy related pathways.

Pathway name^a^	Pathway ID	Con versus A/R			A/R versus DZ			DZ versus DZ5HD		
		DEGs with pathway annotation (335)	*P* value^b^	*Q* value^c^	DEGs with pathway annotation (467)	*P* value^b^	*Q* value^c^	DEGs with pathway annotation (361)	*P* value^b^	*Q* value^c^
Proteasome	ko03050	14 (4.18%)	1.605768*e* − 11	1.686056*e* − 09	20 (4.28%)	2.141436*e* − 16	2.323458*e* − 14	16 (4.43%)	1.912420*e* − 13	2.055852*e* − 11
Fatty acid metabolism	ko00071	8 (2.39%)	4.058827*e* − 05	6.556567*e* − 04	14 (3%)	2.210798*e* − 09	6.853474*e* − 08	9 (2.49%)	9.205386*e* − 06	2.199064*e* − 04
Peroxisome	ko04146	13 (3.88%)	2.092966*e* − 06	5.494036*e* − 05	17 (3.64%)	1.297428*e* − 07	2.815419*e* − 06	12 (3.32%)	2.463547*e* − 05	4.815115*e* − 04
PPAR signaling pathway	ko03320	14 (4.18%)	4.72289*e* − 07	1.653011*e* − 05	16 (3.43%)	9.881599*e* − 07	1.446076*e* − 05	14 (3.88%)	1.150100*e* − 06	4.121192*e* − 05
TCA cycle	ko00020	7 (2.09%)	4.474838*e* − 06	1.044129*e* − 04	14 (3%)	1.954397*e* − 13	1.060260*e* − 11	12 (3.32%)	4.2606*e* − 12	3.053430*e* − 10

^a^Pathway analysis based on KOBAS server 2.0 [21, 22].

^b^ *P* value in hypergeometric test; *P* < 0.01 is considered as DGEs highly enriched.

^c^The *Q* value is similar to the well-known *P*- value, except it is a measure of significance in terms of the false discovery rate rather than the false positive rate [23].
