# Supplementary material for: Genome-Wide Expression Profiling of Anoxia/Reoxygenation in Rat Cardiomyocytes Uncovers the Role of MitoKATP in Energy Homeostasis
Source: Oxid Med Cell Longev. 2015 Jun 15;2015:756576. doi: 10.1155/2015/756576 (PMC4485557; doi:10.1155/2015/756576)
Supplement: Supplementary file 1 — Excel 1: Raw data of the expression of all the gene (4 groups × 3 replicates of the sequencing) . Excel 2: Significant differently expressed genes from Control versus A/R. Excel 3: Significant differently expressed genes from A/R versus DZ. Excel 4: Significant differently expressed genes from DZ versus DZ5HD. Supplementary Figure 1: Assessment of the DGE saturation degree. Supplementary Table 1: The raw data of DGE profile of 12 sequencings. Supplementary Table 2: DEGs significantly enriched GO terms from Control vs A/R (P<0.05). Supplementary Table 3: DEGs significantly enriched GO terms from A/R vs DZ (P<0.05). Supplementary Table 4: DEGs significantly enriched GO terms from DZ vs DZ5HD (P<0.05). Supplementary Table 5: Significantly enriched pathways for DEGs from Con vs A/R (P<0.01). Supplementary Table 6: Significantly enriched pathways for DEGs from A/R vs DZ (P<0.01). Supplementary Table 7: Significantly enriched pathways for DEGs from DZ vs DZ5HD (P<0.01). [file 756576.f1.zip › Supplementary Figure and Tables 2014.12.7.docx]

**Supplementary Material**


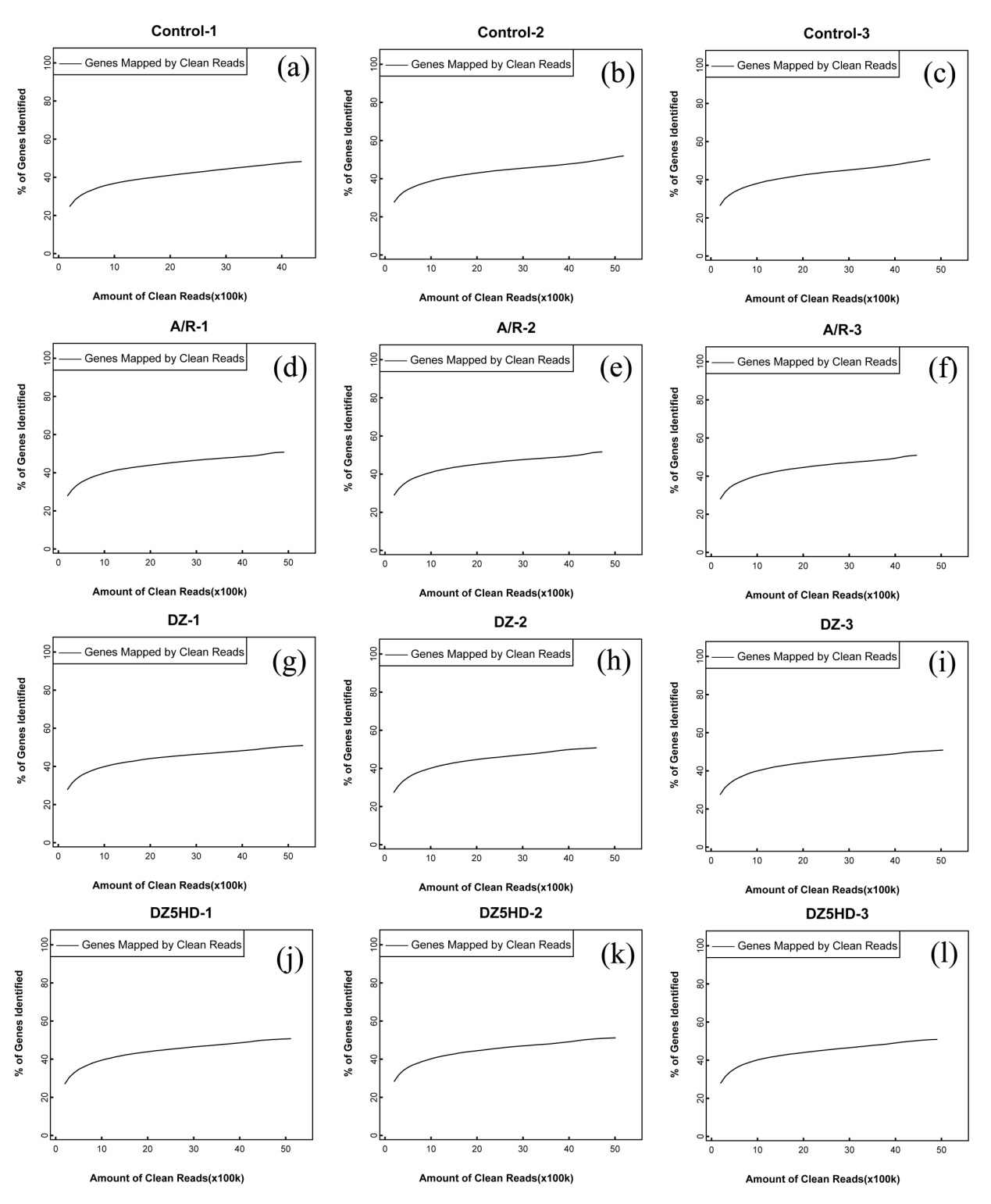


FIGURE 1: Assessment of the DGE saturation degree. Each panel presented the relationship between the percentage of genes identified and total reads number in the library of cardiomyocytes for 4 groups. (a-c): 3 replicates of Con; (d-f): 3 replicates of A/R; (g-i):3 replicates of DZ; (j-l): 3 replicates of DZ5HD.

TABLE 1: The raw data of DGE profile of 12 sequencings.

| **#library** | **Original reads number** | **Original bases number** | **Modified reads number** | **Modified reads rate (%)** | **Modified bases number** | **Low-quality reads number** | **Low-quality reads rate (%)** | **Adapter polluted read number** | **Adapter polluted read rate (%)** | **Original Q30 bases rate (%) *** | **Modified Q30 bases rate (%) #** |
| --- | --- | --- | --- | --- | --- | --- | --- | --- | --- | --- | --- |
| Con-1 | 4428904 | 2.17E+08 | 4349524 | 98.20768 | 2.13E+08 | 69818 | 1.576417 | 9562 | 0.2159 | 96.38245 | 97.05078 |
| Con-2 | 5273434 | 2.58E+08 | 5182471 | 98.27507 | 2.54E+08 | 84017 | 1.593212 | 6946 | 0.131717 | 96.51942 | 97.18693 |
| Con-3 | 4843549 | 2.37E+08 | 4759938 | 98.27377 | 2.33E+08 | 77950 | 1.609357 | 5661 | 0.116877 | 96.48394 | 97.16013 |
| A/R-1 | 5411339 | 2.65E+08 | 5313884 | 98.19906 | 2.6E+08 | 86754 | 1.603189 | 10701 | 0.197751 | 96.50968 | 97.18695 |
| A/R-2 | 4681853 | 2.29E+08 | 4601019 | 98.27346 | 2.25E+08 | 72408 | 1.546567 | 8426 | 0.179971 | 96.57584 | 97.22726 |
| A/R-3 | 5130526 | 2.51E+08 | 5037962 | 98.19582 | 2.47E+08 | 83255 | 1.622738 | 9309 | 0.181443 | 96.47113 | 97.1561 |
| DZ-1 | 5201278 | 2.55E+08 | 5107381 | 98.19473 | 2.5E+08 | 84161 | 1.618083 | 9736 | 0.187185 | 96.4918 | 97.17037 |
| DZ-2 | 5093970 | 2.5E+08 | 5005144 | 98.25625 | 2.45E+08 | 82218 | 1.614026 | 6608 | 0.129722 | 96.53935 | 97.21706 |
| DZ-3 | 4990351 | 2.45E+08 | 4902594 | 98.24147 | 2.4E+08 | 78321 | 1.569449 | 9436 | 0.189085 | 96.54374 | 97.21048 |
| DZ5HD-1 | 4989432 | 2.44E+08 | 4900051 | 98.20859 | 2.4E+08 | 82758 | 1.658666 | 6623 | 0.132741 | 96.48072 | 97.17526 |
| DZ5HD-2 | 4804141 | 2.35E+08 | 4715480 | 98.15449 | 2.31E+08 | 80995 | 1.685941 | 7666 | 0.159571 | 96.41804 | 97.12671 |
| DZ5HD-3 | 4545776 | 2.23E+08 | 4464409 | 98.21005 | 2.19E+08 | 73531 | 1.617568 | 7836 | 0.17238 | 96.5118 | 97.19399 |

* Original Q30 bases rate(%)：The ratio of quality value >30 (error rate <0.1%) bases and the total bases from the original reads before filtering.

# Modified Q30 bases rate(%)：The ratio of quality value >30 (error rate <0.1%) bases and the total bases from the filtered reads.

TABLE 2: DEGs significantly enriched GO terms from Control vs A/R (*P*<0.05)

| **Ontology** | **Gene Ontology term** | **Cluster frequency** | **Genome frequency of use** | **Corrected P-value** |
| --- | --- | --- | --- | --- |
| Biological Process | single-organism metabolic process | 160 out of 357 genes, 44.8% | 2765 out of 17817 genes, 15.5% | 2.68e-37 |
| Biological Process | metabolic process | 304 out of 357 genes, 85.2% | 9612 out of 17817 genes, 53.9% | 1.09e-33 |
| Biological Process | small molecule metabolic process | 127 out of 357 genes, 35.6% | 2032 out of 17817 genes, 11.4% | 8.02e-31 |
| Biological Process | oxoacid metabolic process | 76 out of 357 genes, 21.3% | 831 out of 17817 genes, 4.7% | 1.15e-26 |
| Biological Process | organic acid metabolic process | 76 out of 357 genes, 21.3% | 843 out of 17817 genes, 4.7% | 2.98e-26 |
| Biological Process | carboxylic acid metabolic process | 74 out of 357 genes, 20.7% | 815 out of 17817 genes, 4.6% | 1.17e-25 |
| Biological Process | primary metabolic process | 259 out of 357 genes, 72.5% | 8217 out of 17817 genes, 46.1% | 1.35e-21 |
| Biological Process | organic substance metabolic process | 268 out of 357 genes, 75.1% | 8725 out of 17817 genes, 49.0% | 2.38e-21 |
| Biological Process | cellular metabolic process | 256 out of 357 genes, 71.7% | 8208 out of 17817 genes, 46.1% | 3.33e-20 |
| Biological Process | organonitrogen compound metabolic process | 62 out of 357 genes, 17.4% | 964 out of 17817 genes, 5.4% | 2.18e-13 |
| Biological Process | lipid metabolic process | 60 out of 357 genes, 16.8% | 967 out of 17817 genes, 5.4% | 3.69e-12 |
| Biological Process | cellular amino acid metabolic process | 35 out of 357 genes, 9.8% | 386 out of 17817 genes, 2.2% | 8.34e-11 |
| Biological Process | monocarboxylic acid metabolic process | 33 out of 357 genes, 9.2% | 380 out of 17817 genes, 2.1% | 1.47e-09 |
| Biological Process | cofactor metabolic process | 27 out of 357 genes, 7.6% | 256 out of 17817 genes, 1.4% | 1.90e-09 |
| Biological Process | coenzyme metabolic process | 24 out of 357 genes, 6.7% | 206 out of 17817 genes, 1.2% | 3.96e-09 |
| Biological Process | signal transduction in response to DNA damage | 18 out of 357 genes, 5.0% | 109 out of 17817 genes, 0.6% | 5.51e-09 |
| Biological Process | cellular lipid metabolic process | 44 out of 357 genes, 12.3% | 688 out of 17817 genes, 3.9% | 9.65e-09 |
| Biological Process | generation of precursor metabolites and energy | 31 out of 357 genes, 8.7% | 365 out of 17817 genes, 2.0% | 1.28e-08 |
| Biological Process | proteasomal protein catabolic process | 21 out of 357 genes, 5.9% | 163 out of 17817 genes, 0.9% | 1.30e-08 |
| Biological Process | phosphorus metabolic process | 81 out of 357 genes, 22.7% | 1902 out of 17817 genes, 10.7% | 2.91e-08 |
| Biological Process | proteasomal ubiquitin-dependent protein catabolic process | 20 out of 357 genes, 5.6% | 159 out of 17817 genes, 0.9% | 6.28e-08 |
| Biological Process | DNA damage response, signal transduction by p53 class mediator | 16 out of 357 genes, 4.5% | 97 out of 17817 genes, 0.5% | 8.61e-08 |
| Biological Process | signal transduction by p53 class mediator | 16 out of 357 genes, 4.5% | 97 out of 17817 genes, 0.5% | 8.61e-08 |
| Biological Process | negative regulation of cell cycle | 21 out of 357 genes, 5.9% | 181 out of 17817 genes, 1.0% | 9.74e-08 |
| Biological Process | cell cycle arrest | 20 out of 357 genes, 5.6% | 164 out of 17817 genes, 0.9% | 1.10e-07 |
| Biological Process | carbohydrate metabolic process | 42 out of 357 genes, 11.8% | 688 out of 17817 genes, 3.9% | 1.27e-07 |
| Biological Process | regulation of ubiquitin-protein ligase activity involved in mitotic cell cycle | 17 out of 357 genes, 4.8% | 116 out of 17817 genes, 0.7% | 1.53e-07 |
| Biological Process | regulation of ubiquitin-protein ligase activity | 17 out of 357 genes, 4.8% | 118 out of 17817 genes, 0.7% | 2.01e-07 |
| Biological Process | regulation of ligase activity | 17 out of 357 genes, 4.8% | 119 out of 17817 genes, 0.7% | 2.31e-07 |
| Biological Process | phosphate-containing compound metabolic process | 77 out of 357 genes, 21.6% | 1848 out of 17817 genes, 10.4% | 2.89e-07 |
| Biological Process | regulation of protein ubiquitination | 17 out of 357 genes, 4.8% | 137 out of 17817 genes, 0.8% | 2.15e-06 |
| Biological Process | nitrogen compound metabolic process | 127 out of 357 genes, 35.6% | 3937 out of 17817 genes, 22.1% | 3.02e-06 |
| Biological Process | ubiquitin-dependent protein catabolic process | 21 out of 357 genes, 5.9% | 218 out of 17817 genes, 1.2% | 3.04e-06 |
| Biological Process | oxidation-reduction process | 27 out of 357 genes, 7.6% | 360 out of 17817 genes, 2.0% | 4.35e-06 |
| Biological Process | protein catabolic process | 27 out of 357 genes, 7.6% | 379 out of 17817 genes, 2.1% | 1.29e-05 |
| Biological Process | S phase | 17 out of 357 genes, 4.8% | 158 out of 17817 genes, 0.9% | 1.91e-05 |
| Biological Process | alcohol metabolic process | 21 out of 357 genes, 5.9% | 243 out of 17817 genes, 1.4% | 2.10e-05 |
| Biological Process | organic hydroxy compound metabolic process | 23 out of 357 genes, 6.4% | 292 out of 17817 genes, 1.6% | 2.54e-05 |
| Biological Process | oxidoreduction coenzyme metabolic process | 11 out of 357 genes, 3.1% | 62 out of 17817 genes, 0.3% | 3.58e-05 |
| Biological Process | energy derivation by oxidation of organic compounds | 21 out of 357 genes, 5.9% | 257 out of 17817 genes, 1.4% | 5.54e-05 |
| Biological Process | proteolysis involved in cellular protein catabolic process | 25 out of 357 genes, 7.0% | 366 out of 17817 genes, 2.1% | 0.00010 |
| Biological Process | cellular protein catabolic process | 25 out of 357 genes, 7.0% | 367 out of 17817 genes, 2.1% | 0.00010 |
| Biological Process | modification-dependent protein catabolic process | 24 out of 357 genes, 6.7% | 345 out of 17817 genes, 1.9% | 0.00013 |
| Biological Process | modification-dependent macromolecule catabolic process | 24 out of 357 genes, 6.7% | 345 out of 17817 genes, 1.9% | 0.00013 |
| Biological Process | single-organism carbohydrate metabolic process | 33 out of 357 genes, 9.2% | 608 out of 17817 genes, 3.4% | 0.00022 |
| Biological Process | regulation of fatty acid metabolic process | 8 out of 357 genes, 2.2% | 34 out of 17817 genes, 0.2% | 0.00027 |
| Biological Process | fatty acid metabolic process | 20 out of 357 genes, 5.6% | 268 out of 17817 genes, 1.5% | 0.00049 |
| Biological Process | aerobic respiration | 7 out of 357 genes, 2.0% | 26 out of 17817 genes, 0.1% | 0.00056 |
| Biological Process | macromolecule catabolic process | 34 out of 357 genes, 9.5% | 680 out of 17817 genes, 3.8% | 0.00095 |
| Biological Process | catabolic process | 65 out of 357 genes, 18.2% | 1770 out of 17817 genes, 9.9% | 0.00101 |
| Biological Process | proteolysis | 26 out of 357 genes, 7.3% | 456 out of 17817 genes, 2.6% | 0.00182 |
| Biological Process | regulation of lipid metabolic process | 12 out of 357 genes, 3.4% | 110 out of 17817 genes, 0.6% | 0.00206 |
| Biological Process | hexose metabolic process | 26 out of 357 genes, 7.3% | 461 out of 17817 genes, 2.6% | 0.00222 |
| Biological Process | dicarboxylic acid metabolic process | 9 out of 357 genes, 2.5% | 61 out of 17817 genes, 0.3% | 0.00319 |
| Biological Process | interphase | 17 out of 357 genes, 4.8% | 227 out of 17817 genes, 1.3% | 0.00342 |
| Biological Process | monosaccharide metabolic process | 26 out of 357 genes, 7.3% | 475 out of 17817 genes, 2.7% | 0.00384 |
| Biological Process | cellular nitrogen compound metabolic process | 106 out of 357 genes, 29.7% | 3532 out of 17817 genes, 19.8% | 0.00440 |
| Biological Process | cellular catabolic process | 52 out of 357 genes, 14.6% | 1367 out of 17817 genes, 7.7% | 0.00547 |
| Biological Process | cellular response to stress | 31 out of 357 genes, 8.7% | 644 out of 17817 genes, 3.6% | 0.00635 |
| Biological Process | regulation of cellular ketone metabolic process | 8 out of 357 genes, 2.2% | 51 out of 17817 genes, 0.3% | 0.00707 |
| Biological Process | negative regulation of cellular process | 54 out of 357 genes, 15.1% | 1456 out of 17817 genes, 8.2% | 0.00736 |
| Biological Process | organic substance catabolic process | 60 out of 357 genes, 16.8% | 1684 out of 17817 genes, 9.5% | 0.00743 |
| Biological Process | nucleotide metabolic process | 17 out of 357 genes, 4.8% | 243 out of 17817 genes, 1.4% | 0.00850 |
| Biological Process | response to DNA damage stimulus | 24 out of 357 genes, 6.7% | 437 out of 17817 genes, 2.5% | 0.00860 |
| Biological Process | organic cyclic compound metabolic process | 107 out of 357 genes, 30.0% | 3656 out of 17817 genes, 20.5% | 0.01234 |
| Biological Process | intracellular signal transduction | 45 out of 357 genes, 12.6% | 1155 out of 17817 genes, 6.5% | 0.01392 |
| Biological Process | cellular carbohydrate metabolic process | 11 out of 357 genes, 3.1% | 112 out of 17817 genes, 0.6% | 0.01515 |
| Biological Process | ubiquinone metabolic process | 4 out of 357 genes, 1.1% | 9 out of 17817 genes, 0.1% | 0.01809 |
| Biological Process | branched-chain amino acid metabolic process | 4 out of 357 genes, 1.1% | 9 out of 17817 genes, 0.1% | 0.01809 |
| Biological Process | cellular macromolecule catabolic process | 29 out of 357 genes, 8.1% | 617 out of 17817 genes, 3.5% | 0.01984 |
| Biological Process | negative regulation of biological process | 60 out of 357 genes, 16.8% | 1740 out of 17817 genes, 9.8% | 0.02002 |
| Biological Process | response to stress | 68 out of 357 genes, 19.0% | 2072 out of 17817 genes, 11.6% | 0.02551 |
| Biological Process | sulfur compound metabolic process | 12 out of 357 genes, 3.4% | 141 out of 17817 genes, 0.8% | 0.02683 |
| Biological Process | negative regulation of lipid metabolic process | 4 out of 357 genes, 1.1% | 10 out of 17817 genes, 0.1% | 0.02968 |
| Biological Process | protein homooligomerization | 9 out of 357 genes, 2.5% | 81 out of 17817 genes, 0.5% | 0.03383 |
| Biological Process | regulation of cell cycle | 25 out of 357 genes, 7.0% | 508 out of 17817 genes, 2.9% | 0.03555 |
| Biological Process | cellular amide metabolic process | 8 out of 357 genes, 2.2% | 64 out of 17817 genes, 0.4% | 0.03922 |
| Biological Process | regulation of metabolic process | 102 out of 357 genes, 28.6% | 3542 out of 17817 genes, 19.9% | 0.04313 |
| Biological Process | multicellular organism growth | 8 out of 357 genes, 2.2% | 66 out of 17817 genes, 0.4% | 0.04915 |
| Cellular Component | intracellular part | 338 out of 362 genes, 93.4% | 13253 out of 17912 genes, 74.0% | 8.36e-20 |
| Cellular Component | intracellular | 338 out of 362 genes, 93.4% | 13314 out of 17912 genes, 74.3% | 2.94e-19 |
| Cellular Component | cell | 342 out of 362 genes, 94.5% | 14428 out of 17912 genes, 80.5% | 1.76e-12 |
| Cellular Component | cell part | 342 out of 362 genes, 94.5% | 14428 out of 17912 genes, 80.5% | 1.76e-12 |
| Cellular Component | intracellular membrane-bounded organelle | 267 out of 362 genes, 73.8% | 9854 out of 17912 genes, 55.0% | 1.11e-11 |
| Cellular Component | membrane-bounded organelle | 267 out of 362 genes, 73.8% | 9953 out of 17912 genes, 55.6% | 5.22e-11 |
| Cellular Component | mitochondrial part | 44 out of 362 genes, 12.2% | 654 out of 17912 genes, 3.7% | 3.22e-10 |
| Cellular Component | mitochondrion | 44 out of 362 genes, 12.2% | 660 out of 17912 genes, 3.7% | 4.38e-10 |
| Cellular Component | cytoplasmic part | 182 out of 362 genes, 50.3% | 5962 out of 17912 genes, 33.3% | 1.58e-09 |
| Cellular Component | cytoplasm | 182 out of 362 genes, 50.3% | 5986 out of 17912 genes, 33.4% | 2.32e-09 |
| Cellular Component | proteasome complex | 11 out of 362 genes, 3.0% | 54 out of 17912 genes, 0.3% | 1.14e-06 |
| Cellular Component | intracellular organelle | 278 out of 362 genes, 76.8% | 11325 out of 17912 genes, 63.2% | 2.08e-06 |
| Cellular Component | organelle | 278 out of 362 genes, 76.8% | 11431 out of 17912 genes, 63.8% | 7.46e-06 |
| Cellular Component | organelle envelope | 39 out of 362 genes, 10.8% | 885 out of 17912 genes, 4.9% | 0.00055 |
| Cellular Component | envelope | 39 out of 362 genes, 10.8% | 895 out of 17912 genes, 5.0% | 0.00072 |
| Cellular Component | proteasome accessory complex | 5 out of 362 genes, 1.4% | 21 out of 17912 genes, 0.1% | 0.00674 |
| Cellular Component | lytic vacuole | 17 out of 362 genes, 4.7% | 283 out of 17912 genes, 1.6% | 0.00893 |
| Cellular Component | pigment granule | 10 out of 362 genes, 2.8% | 110 out of 17912 genes, 0.6% | 0.01035 |
| Cellular Component | proteasome core complex | 4 out of 362 genes, 1.1% | 15 out of 17912 genes, 0.1% | 0.02478 |
| Cellular Component | vacuole | 18 out of 362 genes, 5.0% | 339 out of 17912 genes, 1.9% | 0.02617 |
| Cellular Component | protein serine/threonine phosphatase complex | 6 out of 362 genes, 1.7% | 46 out of 17912 genes, 0.3% | 0.04084 |
| Molecular Function | catalytic activity | 227 out of 360 genes, 63.1% | 6353 out of 17883 genes, 35.5% | 1.61e-24 |
| Molecular Function | oxidoreductase activity | 49 out of 360 genes, 13.6% | 764 out of 17883 genes, 4.3% | 1.33e-10 |
| Molecular Function | isomerase activity | 17 out of 360 genes, 4.7% | 164 out of 17883 genes, 0.9% | 7.90e-06 |
| Molecular Function | phosphatase activity | 21 out of 360 genes, 5.8% | 284 out of 17883 genes, 1.6% | 7.14e-05 |
| Molecular Function | hydrolase activity | 84 out of 360 genes, 23.3% | 2528 out of 17883 genes, 14.1% | 0.00034 |
| Molecular Function | binding | 312 out of 360 genes, 86.7% | 13783 out of 17883 genes, 77.1% | 0.00055 |
| Molecular Function | phosphoric ester hydrolase activity | 22 out of 360 genes, 6.1% | 363 out of 17883 genes, 2.0% | 0.00103 |
| Molecular Function | oxidoreductase activity, acting on the CH-CH group of donors | 9 out of 360 genes, 2.5% | 64 out of 17883 genes, 0.4% | 0.00110 |
| Molecular Function | oxidoreductase activity, acting on CH-OH group of donors | 14 out of 360 genes, 3.9% | 163 out of 17883 genes, 0.9% | 0.00121 |
| Molecular Function | hydrolase activity, acting on ester bonds | 35 out of 360 genes, 9.7% | 772 out of 17883 genes, 4.3% | 0.00144 |
| Molecular Function | oxidoreductase activity, acting on the CH-OH group of donors, NAD or NADP as acceptor | 12 out of 360 genes, 3.3% | 128 out of 17883 genes, 0.7% | 0.00232 |
| Molecular Function | coenzyme binding | 11 out of 360 genes, 3.1% | 114 out of 17883 genes, 0.6% | 0.00412 |
| Molecular Function | oxidoreductase activity, acting on the aldehyde or oxo group of donors, NAD or NADP as acceptor | 6 out of 360 genes, 1.7% | 29 out of 17883 genes, 0.2% | 0.00442 |
| Molecular Function | aldehyde dehydrogenase [NAD(P)+] activity | 4 out of 360 genes, 1.1% | 10 out of 17883 genes, 0.1% | 0.00665 |
| Molecular Function | cofactor binding | 15 out of 360 genes, 4.2% | 223 out of 17883 genes, 1.2% | 0.01049 |
| Molecular Function | oxidoreductase activity, acting on the aldehyde or oxo group of donors | 6 out of 360 genes, 1.7% | 40 out of 17883 genes, 0.2% | 0.02968 |
| Molecular Function | intramolecular transferase activity, phosphotransferases | 4 out of 360 genes, 1.1% | 15 out of 17883 genes, 0.1% | 0.03994 |
| Molecular Function | peptidase activity | 28 out of 360 genes, 7.8% | 667 out of 17883 genes, 3.7% | 0.04557 |
| Molecular Function | intramolecular oxidoreductase activity | 7 out of 360 genes, 1.9% | 61 out of 17883 genes, 0.3% | 0.04679 |

TABLE 3: DEGs significantly enriched GO terms from A/R vs DZ (*P*<0.05)

| **Ontology** | **Gene Ontology term** | **Cluster frequency** | **Genome frequency of use** | **Corrected P-value** |
| --- | --- | --- | --- | --- |
| Biological Process | metabolic process | 479 out of 496 genes, 96.6% | 9612 out of 17817 genes, 53.9% | 7.61e-103 |
| Biological Process | single-organism metabolic process | 252 out of 496 genes, 50.8% | 2765 out of 17817 genes, 15.5% | 1.74e-74 |
| Biological Process | primary metabolic process | 403 out of 496 genes, 81.2% | 8217 out of 17817 genes, 46.1% | 6.47e-57 |
| Biological Process | small molecule metabolic process | 193 out of 496 genes, 38.9% | 2032 out of 17817 genes, 11.4% | 2.72e-55 |
| Biological Process | organic substance metabolic process | 410 out of 496 genes, 82.7% | 8725 out of 17817 genes, 49.0% | 3.42e-53 |
| Biological Process | organic acid metabolic process | 121 out of 496 genes, 24.4% | 843 out of 17817 genes, 4.7% | 3.08e-50 |
| Biological Process | cellular metabolic process | 392 out of 496 genes, 79.0% | 8208 out of 17817 genes, 46.1% | 1.80e-49 |
| Biological Process | carboxylic acid metabolic process | 118 out of 496 genes, 23.8% | 815 out of 17817 genes, 4.6% | 3.06e-49 |
| Biological Process | oxoacid metabolic process | 119 out of 496 genes, 24.0% | 831 out of 17817 genes, 4.7% | 3.46e-49 |
| Biological Process | organonitrogen compound metabolic process | 107 out of 496 genes, 21.6% | 964 out of 17817 genes, 5.4% | 3.84e-33 |
| Biological Process | lipid metabolic process | 107 out of 496 genes, 21.6% | 967 out of 17817 genes, 5.4% | 5.10e-33 |
| Biological Process | phosphorus metabolic process | 143 out of 496 genes, 28.8% | 1902 out of 17817 genes, 10.7% | 7.41e-27 |
| Biological Process | cellular amino acid metabolic process | 60 out of 496 genes, 12.1% | 386 out of 17817 genes, 2.2% | 7.18e-25 |
| Biological Process | phosphate-containing compound metabolic process | 134 out of 496 genes, 27.0% | 1848 out of 17817 genes, 10.4% | 3.09e-23 |
| Biological Process | cellular lipid metabolic process | 71 out of 496 genes, 14.3% | 688 out of 17817 genes, 3.9% | 6.57e-19 |
| Biological Process | coenzyme metabolic process | 39 out of 496 genes, 7.9% | 206 out of 17817 genes, 1.2% | 1.49e-18 |
| Biological Process | nitrogen compound metabolic process | 202 out of 496 genes, 40.7% | 3937 out of 17817 genes, 22.1% | 3.07e-18 |
| Biological Process | cofactor metabolic process | 42 out of 496 genes, 8.5% | 256 out of 17817 genes, 1.4% | 1.20e-17 |
| Biological Process | monocarboxylic acid metabolic process | 47 out of 496 genes, 9.5% | 380 out of 17817 genes, 2.1% | 8.17e-15 |
| Biological Process | generation of precursor metabolites and energy | 45 out of 496 genes, 9.1% | 365 out of 17817 genes, 2.0% | 4.84e-14 |
| Biological Process | dicarboxylic acid metabolic process | 20 out of 496 genes, 4.0% | 61 out of 17817 genes, 0.3% | 1.35e-13 |
| Biological Process | oxidation-reduction process | 44 out of 496 genes, 8.9% | 360 out of 17817 genes, 2.0% | 1.53e-13 |
| Biological Process | signal transduction in response to DNA damage | 25 out of 496 genes, 5.0% | 109 out of 17817 genes, 0.6% | 2.69e-13 |
| Biological Process | aerobic respiration | 14 out of 496 genes, 2.8% | 26 out of 17817 genes, 0.1% | 1.16e-12 |
| Biological Process | DNA damage response, signal transduction by p53 class mediator | 23 out of 496 genes, 4.6% | 97 out of 17817 genes, 0.5% | 1.97e-12 |
| Biological Process | signal transduction by p53 class mediator | 23 out of 496 genes, 4.6% | 97 out of 17817 genes, 0.5% | 1.97e-12 |
| Biological Process | carbohydrate metabolic process | 60 out of 496 genes, 12.1% | 688 out of 17817 genes, 3.9% | 3.86e-12 |
| Biological Process | energy derivation by oxidation of organic compounds | 35 out of 496 genes, 7.1% | 257 out of 17817 genes, 1.4% | 8.31e-12 |
| Biological Process | alcohol metabolic process | 34 out of 496 genes, 6.9% | 243 out of 17817 genes, 1.4% | 9.04e-12 |
| Biological Process | regulation of ligase activity | 24 out of 496 genes, 4.8% | 119 out of 17817 genes, 0.7% | 2.36e-11 |
| Biological Process | S phase | 27 out of 496 genes, 5.4% | 158 out of 17817 genes, 0.9% | 4.01e-11 |
| Biological Process | organic hydroxy compound metabolic process | 36 out of 496 genes, 7.3% | 292 out of 17817 genes, 1.6% | 7.64e-11 |
| Biological Process | proteasomal protein catabolic process | 27 out of 496 genes, 5.4% | 163 out of 17817 genes, 0.9% | 8.84e-11 |
| Biological Process | regulation of ubiquitin-protein ligase activity involved in mitotic cell cycle | 23 out of 496 genes, 4.6% | 116 out of 17817 genes, 0.7% | 1.22e-10 |
| Biological Process | regulation of ubiquitin-protein ligase activity | 23 out of 496 genes, 4.6% | 118 out of 17817 genes, 0.7% | 1.79e-10 |
| Biological Process | proteasomal ubiquitin-dependent protein catabolic process | 26 out of 496 genes, 5.2% | 159 out of 17817 genes, 0.9% | 3.52e-10 |
| Biological Process | cellular nitrogen compound metabolic process | 166 out of 496 genes, 33.5% | 3532 out of 17817 genes, 19.8% | 3.54e-10 |
| Biological Process | oxidoreduction coenzyme metabolic process | 17 out of 496 genes, 3.4% | 62 out of 17817 genes, 0.3% | 7.49e-10 |
| Biological Process | organic cyclic compound metabolic process | 167 out of 496 genes, 33.7% | 3656 out of 17817 genes, 20.5% | 3.49e-09 |
| Biological Process | hexose metabolic process | 43 out of 496 genes, 8.7% | 461 out of 17817 genes, 2.6% | 4.54e-09 |
| Biological Process | regulation of protein ubiquitination | 23 out of 496 genes, 4.6% | 137 out of 17817 genes, 0.8% | 4.77e-09 |
| Biological Process | cell cycle arrest | 25 out of 496 genes, 5.0% | 164 out of 17817 genes, 0.9% | 5.11e-09 |
| Biological Process | negative regulation of cell cycle | 26 out of 496 genes, 5.2% | 181 out of 17817 genes, 1.0% | 7.65e-09 |
| Biological Process | protein catabolic process | 38 out of 496 genes, 7.7% | 379 out of 17817 genes, 2.1% | 9.94e-09 |
| Biological Process | monosaccharide metabolic process | 43 out of 496 genes, 8.7% | 475 out of 17817 genes, 2.7% | 1.22e-08 |
| Biological Process | ubiquitin-dependent protein catabolic process | 28 out of 496 genes, 5.6% | 218 out of 17817 genes, 1.2% | 1.85e-08 |
| Biological Process | fatty acid metabolic process | 31 out of 496 genes, 6.2% | 268 out of 17817 genes, 1.5% | 2.35e-08 |
| Biological Process | interphase | 28 out of 496 genes, 5.6% | 227 out of 17817 genes, 1.3% | 4.91e-08 |
| Biological Process | regulation of fatty acid metabolic process | 12 out of 496 genes, 2.4% | 34 out of 17817 genes, 0.2% | 6.95e-08 |
| Biological Process | single-organism carbohydrate metabolic process | 48 out of 496 genes, 9.7% | 608 out of 17817 genes, 3.4% | 9.27e-08 |
| Biological Process | pyridine nucleotide metabolic process | 14 out of 496 genes, 2.8% | 53 out of 17817 genes, 0.3% | 1.45e-07 |
| Biological Process | nicotinamide nucleotide metabolic process | 14 out of 496 genes, 2.8% | 53 out of 17817 genes, 0.3% | 1.45e-07 |
| Biological Process | NAD metabolic process | 8 out of 496 genes, 1.6% | 12 out of 17817 genes, 0.1% | 1.78e-07 |
| Biological Process | sulfur compound metabolic process | 21 out of 496 genes, 4.2% | 141 out of 17817 genes, 0.8% | 4.19e-07 |
| Biological Process | response to stress | 105 out of 496 genes, 21.2% | 2072 out of 17817 genes, 11.6% | 6.31e-07 |
| Biological Process | cellular response to stress | 48 out of 496 genes, 9.7% | 644 out of 17817 genes, 3.6% | 6.50e-07 |
| Biological Process | pyridine-containing compound metabolic process | 14 out of 496 genes, 2.8% | 59 out of 17817 genes, 0.3% | 6.88e-07 |
| Biological Process | proteolysis involved in cellular protein catabolic process | 34 out of 496 genes, 6.9% | 366 out of 17817 genes, 2.1% | 9.84e-07 |
| Biological Process | cellular protein catabolic process | 34 out of 496 genes, 6.9% | 367 out of 17817 genes, 2.1% | 1.05e-06 |
| Biological Process | regulation of cellular ketone metabolic process | 13 out of 496 genes, 2.6% | 51 out of 17817 genes, 0.3% | 1.09e-06 |
| Biological Process | modification-dependent protein catabolic process | 32 out of 496 genes, 6.5% | 345 out of 17817 genes, 1.9% | 3.25e-06 |
| Biological Process | modification-dependent macromolecule catabolic process | 32 out of 496 genes, 6.5% | 345 out of 17817 genes, 1.9% | 3.25e-06 |
| Biological Process | cellular process | 430 out of 496 genes, 86.7% | 13596 out of 17817 genes, 76.3% | 3.97e-06 |
| Biological Process | nucleotide metabolic process | 26 out of 496 genes, 5.2% | 243 out of 17817 genes, 1.4% | 5.39e-06 |
| Biological Process | cellular respiration | 17 out of 496 genes, 3.4% | 105 out of 17817 genes, 0.6% | 5.50e-06 |
| Biological Process | acylglycerol metabolic process | 16 out of 496 genes, 3.2% | 96 out of 17817 genes, 0.5% | 1.01e-05 |
| Biological Process | macromolecule catabolic process | 47 out of 496 genes, 9.5% | 680 out of 17817 genes, 3.8% | 1.10e-05 |
| Biological Process | regulation of lipid metabolic process | 17 out of 496 genes, 3.4% | 110 out of 17817 genes, 0.6% | 1.14e-05 |
| Biological Process | neutral lipid metabolic process | 16 out of 496 genes, 3.2% | 97 out of 17817 genes, 0.5% | 1.18e-05 |
| Biological Process | organophosphate metabolic process | 64 out of 496 genes, 12.9% | 1091 out of 17817 genes, 6.1% | 1.36e-05 |
| Biological Process | cellular carbohydrate metabolic process | 17 out of 496 genes, 3.4% | 112 out of 17817 genes, 0.6% | 1.50e-05 |
| Biological Process | negative regulation of cellular process | 78 out of 496 genes, 15.7% | 1456 out of 17817 genes, 8.2% | 1.59e-05 |
| Biological Process | cellular amide metabolic process | 13 out of 496 genes, 2.6% | 64 out of 17817 genes, 0.4% | 2.16e-05 |
| Biological Process | proteolysis | 36 out of 496 genes, 7.3% | 456 out of 17817 genes, 2.6% | 2.39e-05 |
| Biological Process | protein metabolic process | 175 out of 496 genes, 35.3% | 4340 out of 17817 genes, 24.4% | 2.51e-05 |
| Biological Process | cellular aromatic compound metabolic process | 146 out of 496 genes, 29.4% | 3457 out of 17817 genes, 19.4% | 4.00e-05 |
| Biological Process | polysaccharide metabolic process | 12 out of 496 genes, 2.4% | 57 out of 17817 genes, 0.3% | 5.01e-05 |
| Biological Process | cellular polysaccharide metabolic process | 12 out of 496 genes, 2.4% | 57 out of 17817 genes, 0.3% | 5.01e-05 |
| Biological Process | steroid metabolic process | 24 out of 496 genes, 4.8% | 238 out of 17817 genes, 1.3% | 6.67e-05 |
| Biological Process | cellular macromolecule catabolic process | 42 out of 496 genes, 8.5% | 617 out of 17817 genes, 3.5% | 0.00010 |
| Biological Process | catabolic process | 87 out of 496 genes, 17.5% | 1770 out of 17817 genes, 9.9% | 0.00010 |
| Biological Process | sterol metabolic process | 16 out of 496 genes, 3.2% | 113 out of 17817 genes, 0.6% | 0.00011 |
| Biological Process | nucleobase-containing compound metabolic process | 139 out of 496 genes, 28.0% | 3299 out of 17817 genes, 18.5% | 0.00011 |
| Biological Process | heterocycle metabolic process | 142 out of 496 genes, 28.6% | 3415 out of 17817 genes, 19.2% | 0.00018 |
| Biological Process | cellular glucan metabolic process | 11 out of 496 genes, 2.2% | 53 out of 17817 genes, 0.3% | 0.00021 |
| Biological Process | glucan metabolic process | 11 out of 496 genes, 2.2% | 53 out of 17817 genes, 0.3% | 0.00021 |
| Biological Process | one-carbon metabolic process | 15 out of 496 genes, 3.0% | 106 out of 17817 genes, 0.6% | 0.00027 |
| Biological Process | alpha-amino acid metabolic process | 17 out of 496 genes, 3.4% | 136 out of 17817 genes, 0.8% | 0.00028 |
| Biological Process | organic substance catabolic process | 82 out of 496 genes, 16.5% | 1684 out of 17817 genes, 9.5% | 0.00040 |
| Biological Process | negative regulation of biological process | 84 out of 496 genes, 16.9% | 1740 out of 17817 genes, 9.8% | 0.00040 |
| Biological Process | glycerolipid metabolic process | 22 out of 496 genes, 4.4% | 227 out of 17817 genes, 1.3% | 0.00047 |
| Biological Process | cell cycle | 59 out of 496 genes, 11.9% | 1072 out of 17817 genes, 6.0% | 0.00047 |
| Biological Process | cell cycle process | 54 out of 496 genes, 10.9% | 947 out of 17817 genes, 5.3% | 0.00052 |
| Biological Process | glycogen metabolic process | 9 out of 496 genes, 1.8% | 37 out of 17817 genes, 0.2% | 0.00067 |
| Biological Process | energy reserve metabolic process | 9 out of 496 genes, 1.8% | 37 out of 17817 genes, 0.2% | 0.00067 |
| Biological Process | cellular modified amino acid metabolic process | 14 out of 496 genes, 2.8% | 99 out of 17817 genes, 0.6% | 0.00071 |
| Biological Process | small molecule catabolic process | 16 out of 496 genes, 3.2% | 130 out of 17817 genes, 0.7% | 0.00078 |
| Biological Process | single-organism catabolic process | 16 out of 496 genes, 3.2% | 130 out of 17817 genes, 0.7% | 0.00078 |
| Biological Process | response to DNA damage stimulus | 32 out of 496 genes, 6.5% | 437 out of 17817 genes, 2.5% | 0.00080 |
| Biological Process | cellular catabolic process | 69 out of 496 genes, 13.9% | 1367 out of 17817 genes, 7.7% | 0.00112 |
| Biological Process | carboxylic acid catabolic process | 15 out of 496 genes, 3.0% | 119 out of 17817 genes, 0.7% | 0.00128 |
| Biological Process | peptide metabolic process | 8 out of 496 genes, 1.6% | 30 out of 17817 genes, 0.2% | 0.00135 |
| Biological Process | organic acid catabolic process | 15 out of 496 genes, 3.0% | 121 out of 17817 genes, 0.7% | 0.00158 |
| Biological Process | regulation of metabolic process | 142 out of 496 genes, 28.6% | 3542 out of 17817 genes, 19.9% | 0.00159 |
| Biological Process | mitotic cell cycle | 34 out of 496 genes, 6.9% | 501 out of 17817 genes, 2.8% | 0.00201 |
| Biological Process | branched-chain amino acid metabolic process | 5 out of 496 genes, 1.0% | 9 out of 17817 genes, 0.1% | 0.00219 |
| Biological Process | negative regulation of apoptotic process | 26 out of 496 genes, 5.2% | 337 out of 17817 genes, 1.9% | 0.00349 |
| Biological Process | membrane lipid metabolic process | 15 out of 496 genes, 3.0% | 129 out of 17817 genes, 0.7% | 0.00359 |
| Biological Process | negative regulation of programmed cell death | 26 out of 496 genes, 5.2% | 338 out of 17817 genes, 1.9% | 0.00368 |
| Biological Process | negative regulation of cell death | 26 out of 496 genes, 5.2% | 338 out of 17817 genes, 1.9% | 0.00368 |
| Biological Process | triglyceride metabolic process | 10 out of 496 genes, 2.0% | 57 out of 17817 genes, 0.3% | 0.00397 |
| Biological Process | nucleobase-containing small molecule metabolic process | 49 out of 496 genes, 9.9% | 895 out of 17817 genes, 5.0% | 0.00572 |
| Biological Process | regulation of cell cycle | 33 out of 496 genes, 6.7% | 508 out of 17817 genes, 2.9% | 0.00728 |
| Biological Process | regulation of apoptotic process | 36 out of 496 genes, 7.3% | 581 out of 17817 genes, 3.3% | 0.00794 |
| Biological Process | hydrogen peroxide metabolic process | 8 out of 496 genes, 1.6% | 40 out of 17817 genes, 0.2% | 0.01395 |
| Biological Process | macromolecule metabolic process | 236 out of 496 genes, 47.6% | 6829 out of 17817 genes, 38.3% | 0.01517 |
| Biological Process | glutamine family amino acid metabolic process | 10 out of 496 genes, 2.0% | 66 out of 17817 genes, 0.4% | 0.01551 |
| Biological Process | protein homooligomerization | 11 out of 496 genes, 2.2% | 81 out of 17817 genes, 0.5% | 0.01696 |
| Biological Process | leucine metabolic process | 3 out of 496 genes, 0.6% | 3 out of 17817 genes, 0.0% | 0.02500 |
| Biological Process | aspartate family amino acid metabolic process | 7 out of 496 genes, 1.4% | 32 out of 17817 genes, 0.2% | 0.02663 |
| Biological Process | cholesterol metabolic process | 9 out of 496 genes, 1.8% | 58 out of 17817 genes, 0.3% | 0.03447 |
| Biological Process | transmembrane receptor protein tyrosine kinase signaling pathway | 30 out of 496 genes, 6.0% | 477 out of 17817 genes, 2.7% | 0.03544 |
| Biological Process | fatty acid catabolic process | 10 out of 496 genes, 2.0% | 73 out of 17817 genes, 0.4% | 0.03840 |
| Biological Process | acyl-CoA metabolic process | 8 out of 496 genes, 1.6% | 46 out of 17817 genes, 0.3% | 0.04088 |
| Biological Process | thioester metabolic process | 8 out of 496 genes, 1.6% | 46 out of 17817 genes, 0.3% | 0.04088 |
| Biological Process | monocarboxylic acid catabolic process | 10 out of 496 genes, 2.0% | 74 out of 17817 genes, 0.4% | 0.04331 |
| Biological Process | ethanol metabolic process | 4 out of 496 genes, 0.8% | 8 out of 17817 genes, 0.0% | 0.04431 |
| Biological Process | lysine metabolic process | 4 out of 496 genes, 0.8% | 8 out of 17817 genes, 0.0% | 0.04431 |
| Cellular Component | intracellular part | 447 out of 470 genes, 95.1% | 13253 out of 17912 genes, 74.0% | 3.16e-32 |
| Cellular Component | intracellular | 447 out of 470 genes, 95.1% | 13314 out of 17912 genes, 74.3% | 1.88e-31 |
| Cellular Component | mitochondrial part | 73 out of 470 genes, 15.5% | 654 out of 17912 genes, 3.7% | 4.58e-24 |
| Cellular Component | mitochondrion | 73 out of 470 genes, 15.5% | 660 out of 17912 genes, 3.7% | 8.09e-24 |
| Cellular Component | cell | 454 out of 470 genes, 96.6% | 14428 out of 17912 genes, 80.5% | 1.27e-23 |
| Cellular Component | cell part | 454 out of 470 genes, 96.6% | 14428 out of 17912 genes, 80.5% | 1.27e-23 |
| Cellular Component | cytoplasm | 257 out of 470 genes, 54.7% | 5986 out of 17912 genes, 33.4% | 1.13e-19 |
| Cellular Component | cytoplasmic part | 256 out of 470 genes, 54.5% | 5962 out of 17912 genes, 33.3% | 1.51e-19 |
| Cellular Component | intracellular membrane-bounded organelle | 357 out of 470 genes, 76.0% | 9854 out of 17912 genes, 55.0% | 1.89e-19 |
| Cellular Component | membrane-bounded organelle | 357 out of 470 genes, 76.0% | 9953 out of 17912 genes, 55.6% | 1.78e-18 |
| Cellular Component | organelle envelope | 64 out of 470 genes, 13.6% | 885 out of 17912 genes, 4.9% | 2.20e-11 |
| Cellular Component | envelope | 64 out of 470 genes, 13.6% | 895 out of 17912 genes, 5.0% | 3.67e-11 |
| Cellular Component | intracellular organelle | 369 out of 470 genes, 78.5% | 11325 out of 17912 genes, 63.2% | 5.02e-11 |
| Cellular Component | proteasome complex | 16 out of 470 genes, 3.4% | 54 out of 17912 genes, 0.3% | 5.12e-11 |
| Cellular Component | organelle | 369 out of 470 genes, 78.5% | 11431 out of 17912 genes, 63.8% | 3.24e-10 |
| Cellular Component | organelle part | 230 out of 470 genes, 48.9% | 6284 out of 17912 genes, 35.1% | 4.46e-08 |
| Cellular Component | intracellular organelle part | 209 out of 470 genes, 44.5% | 5689 out of 17912 genes, 31.8% | 5.37e-07 |
| Cellular Component | proteasome accessory complex | 8 out of 470 genes, 1.7% | 21 out of 17912 genes, 0.1% | 4.94e-06 |
| Cellular Component | protein complex | 105 out of 470 genes, 22.3% | 2447 out of 17912 genes, 13.7% | 2.28e-05 |
| Cellular Component | organelle membrane | 82 out of 470 genes, 17.4% | 1767 out of 17912 genes, 9.9% | 3.17e-05 |
| Cellular Component | organelle inner membrane | 34 out of 470 genes, 7.2% | 519 out of 17912 genes, 2.9% | 0.00015 |
| Cellular Component | mitochondrial envelope | 29 out of 470 genes, 6.2% | 427 out of 17912 genes, 2.4% | 0.00049 |
| Cellular Component | pigment granule | 13 out of 470 genes, 2.8% | 110 out of 17912 genes, 0.6% | 0.00096 |
| Cellular Component | protein-lipid complex | 7 out of 470 genes, 1.5% | 30 out of 17912 genes, 0.2% | 0.00153 |
| Cellular Component | plasma lipoprotein particle | 7 out of 470 genes, 1.5% | 30 out of 17912 genes, 0.2% | 0.00153 |
| Cellular Component | lytic vacuole | 21 out of 470 genes, 4.5% | 283 out of 17912 genes, 1.6% | 0.00308 |
| Cellular Component | protein serine/threonine phosphatase complex | 8 out of 470 genes, 1.7% | 46 out of 17912 genes, 0.3% | 0.00356 |
| Cellular Component | proteasome core complex | 5 out of 470 genes, 1.1% | 15 out of 17912 genes, 0.1% | 0.00455 |
| Cellular Component | vacuole | 22 out of 470 genes, 4.7% | 339 out of 17912 genes, 1.9% | 0.01488 |
| Cellular Component | extracellular space | 7 out of 470 genes, 1.5% | 45 out of 17912 genes, 0.3% | 0.02429 |
| Molecular Function | catalytic activity | 357 out of 487 genes, 73.3% | 6353 out of 17883 genes, 35.5% | 5.70e-64 |
| Molecular Function | oxidoreductase activity | 73 out of 487 genes, 15.0% | 764 out of 17883 genes, 4.3% | 9.21e-19 |
| Molecular Function | phosphatase activity | 37 out of 487 genes, 7.6% | 284 out of 17883 genes, 1.6% | 6.59e-13 |
| Molecular Function | transferase activity | 120 out of 487 genes, 24.6% | 2236 out of 17883 genes, 12.5% | 2.16e-11 |
| Molecular Function | hydrolase activity | 128 out of 487 genes, 26.3% | 2528 out of 17883 genes, 14.1% | 1.75e-10 |
| Molecular Function | cofactor binding | 29 out of 487 genes, 6.0% | 223 out of 17883 genes, 1.2% | 7.73e-10 |
| Molecular Function | phosphoric ester hydrolase activity | 37 out of 487 genes, 7.6% | 363 out of 17883 genes, 2.0% | 1.38e-09 |
| Molecular Function | hydrolase activity, acting on ester bonds | 57 out of 487 genes, 11.7% | 772 out of 17883 genes, 4.3% | 1.86e-09 |
| Molecular Function | oxidoreductase activity, acting on the CH-CH group of donors | 14 out of 487 genes, 2.9% | 64 out of 17883 genes, 0.4% | 3.73e-07 |
| Molecular Function | transferase activity, transferring phosphorus-containing groups | 67 out of 487 genes, 13.8% | 1140 out of 17883 genes, 6.4% | 5.05e-07 |
| Molecular Function | oxidoreductase activity, acting on CH-OH group of donors | 21 out of 487 genes, 4.3% | 163 out of 17883 genes, 0.9% | 9.63e-07 |
| Molecular Function | coenzyme binding | 17 out of 487 genes, 3.5% | 114 out of 17883 genes, 0.6% | 3.26e-06 |
| Molecular Function | oxidoreductase activity, acting on the CH-OH group of donors, NAD or NADP as acceptor | 17 out of 487 genes, 3.5% | 128 out of 17883 genes, 0.7% | 1.89e-05 |
| Molecular Function | isocitrate dehydrogenase activity | 5 out of 487 genes, 1.0% | 6 out of 17883 genes, 0.0% | 2.28e-05 |
| Molecular Function | adenyl nucleotide binding | 80 out of 487 genes, 16.4% | 1614 out of 17883 genes, 9.0% | 2.50e-05 |
| Molecular Function | adenyl ribonucleotide binding | 79 out of 487 genes, 16.2% | 1602 out of 17883 genes, 9.0% | 3.78e-05 |
| Molecular Function | peptidase activity, acting on L-amino acid peptides | 42 out of 487 genes, 8.6% | 656 out of 17883 genes, 3.7% | 7.15e-05 |
| Molecular Function | peptidase activity | 42 out of 487 genes, 8.6% | 667 out of 17883 genes, 3.7% | 0.00011 |
| Molecular Function | isomerase activity | 18 out of 487 genes, 3.7% | 164 out of 17883 genes, 0.9% | 0.00014 |
| Molecular Function | small molecule binding | 107 out of 487 genes, 22.0% | 2487 out of 17883 genes, 13.9% | 0.00017 |
| Molecular Function | oxidoreductase activity, acting on the aldehyde or oxo group of donors, NAD or NADP as acceptor | 8 out of 487 genes, 1.6% | 29 out of 17883 genes, 0.2% | 0.00019 |
| Molecular Function | acyl-CoA dehydrogenase activity | 6 out of 487 genes, 1.2% | 14 out of 17883 genes, 0.1% | 0.00026 |
| Molecular Function | kinase activity | 45 out of 487 genes, 9.2% | 790 out of 17883 genes, 4.4% | 0.00066 |
| Molecular Function | malate dehydrogenase activity | 4 out of 487 genes, 0.8% | 5 out of 17883 genes, 0.0% | 0.00070 |
| Molecular Function | aldehyde dehydrogenase [NAD(P)+] activity | 5 out of 487 genes, 1.0% | 10 out of 17883 genes, 0.1% | 0.00087 |
| Molecular Function | nucleotide binding | 100 out of 487 genes, 20.5% | 2360 out of 17883 genes, 13.2% | 0.00088 |
| Molecular Function | nucleoside phosphate binding | 100 out of 487 genes, 20.5% | 2360 out of 17883 genes, 13.2% | 0.00088 |
| Molecular Function | oxidoreductase activity, acting on the aldehyde or oxo group of donors | 8 out of 487 genes, 1.6% | 40 out of 17883 genes, 0.2% | 0.00270 |
| Molecular Function | phosphoprotein phosphatase activity | 16 out of 487 genes, 3.3% | 164 out of 17883 genes, 0.9% | 0.00287 |
| Molecular Function | antioxidant activity | 9 out of 487 genes, 1.8% | 57 out of 17883 genes, 0.3% | 0.00570 |
| Molecular Function | purine nucleotide binding | 84 out of 487 genes, 17.2% | 1999 out of 17883 genes, 11.2% | 0.00869 |
| Molecular Function | ion binding | 142 out of 487 genes, 29.2% | 3841 out of 17883 genes, 21.5% | 0.00871 |
| Molecular Function | intramolecular transferase activity, phosphotransferases | 5 out of 487 genes, 1.0% | 15 out of 17883 genes, 0.1% | 0.00931 |
| Molecular Function | phosphotransferase activity, alcohol group as acceptor | 35 out of 487 genes, 7.2% | 620 out of 17883 genes, 3.5% | 0.01075 |
| Molecular Function | binding | 410 out of 487 genes, 84.2% | 13783 out of 17883 genes, 77.1% | 0.01403 |
| Molecular Function | ribonucleotide binding | 82 out of 487 genes, 16.8% | 1982 out of 17883 genes, 11.1% | 0.01882 |
| Molecular Function | purine ribonucleotide binding | 82 out of 487 genes, 16.8% | 1982 out of 17883 genes, 11.1% | 0.01882 |
| Molecular Function | carboxylic acid binding | 12 out of 487 genes, 2.5% | 118 out of 17883 genes, 0.7% | 0.02405 |
| Molecular Function | exopeptidase activity | 10 out of 487 genes, 2.1% | 87 out of 17883 genes, 0.5% | 0.03320 |

TABLE 4: DEGs significantly enriched GO terms from DZ vs DZ5HD (*P*<0.05)

| **Ontology** | **Gene Ontology term** | **Cluster frequency** | **Genome frequency of use** | **Corrected P-value** |
| --- | --- | --- | --- | --- |
| Biological Process | metabolic process | 369 out of 381 genes, 96.9% | 9612 out of 17817 genes, 53.9% | 9.60e-80 |
| Biological Process | single-organism metabolic process | 189 out of 381 genes, 49.6% | 2765 out of 17817 genes, 15.5% | 6.43e-53 |
| Biological Process | organic acid metabolic process | 108 out of 381 genes, 28.3% | 843 out of 17817 genes, 4.7% | 1.76e-51 |
| Biological Process | carboxylic acid metabolic process | 106 out of 381 genes, 27.8% | 815 out of 17817 genes, 4.6% | 4.76e-51 |
| Biological Process | oxoacid metabolic process | 106 out of 381 genes, 27.8% | 831 out of 17817 genes, 4.7% | 3.33e-50 |
| Biological Process | small molecule metabolic process | 148 out of 381 genes, 38.8% | 2032 out of 17817 genes, 11.4% | 1.32e-41 |
| Biological Process | organic substance metabolic process | 311 out of 381 genes, 81.6% | 8725 out of 17817 genes, 49.0% | 1.33e-37 |
| Biological Process | cellular metabolic process | 298 out of 381 genes, 78.2% | 8208 out of 17817 genes, 46.1% | 1.80e-35 |
| Biological Process | primary metabolic process | 294 out of 381 genes, 77.2% | 8217 out of 17817 genes, 46.1% | 7.14e-33 |
| Biological Process | organonitrogen compound metabolic process | 83 out of 381 genes, 21.8% | 964 out of 17817 genes, 5.4% | 1.79e-25 |
| Biological Process | lipid metabolic process | 82 out of 381 genes, 21.5% | 967 out of 17817 genes, 5.4% | 1.16e-24 |
| Biological Process | cellular amino acid metabolic process | 48 out of 381 genes, 12.6% | 386 out of 17817 genes, 2.2% | 3.56e-20 |
| Biological Process | phosphorus metabolic process | 108 out of 381 genes, 28.3% | 1902 out of 17817 genes, 10.7% | 3.73e-19 |
| Biological Process | cellular lipid metabolic process | 61 out of 381 genes, 16.0% | 688 out of 17817 genes, 3.9% | 1.76e-18 |
| Biological Process | phosphate-containing compound metabolic process | 101 out of 381 genes, 26.5% | 1848 out of 17817 genes, 10.4% | 2.12e-16 |
| Biological Process | monocarboxylic acid metabolic process | 43 out of 381 genes, 11.3% | 380 out of 17817 genes, 2.1% | 3.04e-16 |
| Biological Process | nitrogen compound metabolic process | 155 out of 381 genes, 40.7% | 3937 out of 17817 genes, 22.1% | 1.49e-13 |
| Biological Process | cofactor metabolic process | 31 out of 381 genes, 8.1% | 256 out of 17817 genes, 1.4% | 5.70e-12 |
| Biological Process | coenzyme metabolic process | 27 out of 381 genes, 7.1% | 206 out of 17817 genes, 1.2% | 4.91e-11 |
| Biological Process | aerobic respiration | 12 out of 381 genes, 3.1% | 26 out of 17817 genes, 0.1% | 6.03e-11 |
| Biological Process | oxidation-reduction process | 35 out of 381 genes, 9.2% | 360 out of 17817 genes, 2.0% | 7.88e-11 |
| Biological Process | generation of precursor metabolites and energy | 35 out of 381 genes, 9.2% | 365 out of 17817 genes, 2.0% | 1.18e-10 |
| Biological Process | dicarboxylic acid metabolic process | 16 out of 381 genes, 4.2% | 61 out of 17817 genes, 0.3% | 1.25e-10 |
| Biological Process | signal transduction in response to DNA damage | 20 out of 381 genes, 5.2% | 109 out of 17817 genes, 0.6% | 1.63e-10 |
| Biological Process | DNA damage response, signal transduction by p53 class mediator | 19 out of 381 genes, 5.0% | 97 out of 17817 genes, 0.5% | 1.93e-10 |
| Biological Process | signal transduction by p53 class mediator | 19 out of 381 genes, 5.0% | 97 out of 17817 genes, 0.5% | 1.93e-10 |
| Biological Process | energy derivation by oxidation of organic compounds | 29 out of 381 genes, 7.6% | 257 out of 17817 genes, 1.4% | 2.77e-10 |
| Biological Process | fatty acid metabolic process | 29 out of 381 genes, 7.6% | 268 out of 17817 genes, 1.5% | 8.11e-10 |
| Biological Process | regulation of ligase activity | 19 out of 381 genes, 5.0% | 119 out of 17817 genes, 0.7% | 8.87e-09 |
| Biological Process | S phase | 21 out of 381 genes, 5.5% | 158 out of 17817 genes, 0.9% | 2.62e-08 |
| Biological Process | proteasomal ubiquitin-dependent protein catabolic process | 21 out of 381 genes, 5.5% | 159 out of 17817 genes, 0.9% | 2.96e-08 |
| Biological Process | proteasomal protein catabolic process | 21 out of 381 genes, 5.5% | 163 out of 17817 genes, 0.9% | 4.78e-08 |
| Biological Process | regulation of ubiquitin-protein ligase activity involved in mitotic cell cycle | 18 out of 381 genes, 4.7% | 116 out of 17817 genes, 0.7% | 5.18e-08 |
| Biological Process | regulation of ubiquitin-protein ligase activity | 18 out of 381 genes, 4.7% | 118 out of 17817 genes, 0.7% | 6.94e-08 |
| Biological Process | monosaccharide metabolic process | 35 out of 381 genes, 9.2% | 475 out of 17817 genes, 2.7% | 2.12e-07 |
| Biological Process | carbohydrate metabolic process | 43 out of 381 genes, 11.3% | 688 out of 17817 genes, 3.9% | 3.13e-07 |
| Biological Process | ubirquitin-dependent protein catabolic process | 23 out of 381 genes, 6.0% | 218 out of 17817 genes, 1.2% | 3.37e-07 |
| Biological Process | negative regulation of cell cycle | 21 out of 381 genes, 5.5% | 181 out of 17817 genes, 1.0% | 3.48e-07 |
| Biological Process | cell cycle arrest | 20 out of 381 genes, 5.2% | 164 out of 17817 genes, 0.9% | 3.78e-07 |
| Biological Process | cellular respiration | 16 out of 381 genes, 4.2% | 105 out of 17817 genes, 0.6% | 8.22e-07 |
| Biological Process | regulation of protein ubiquitination | 18 out of 381 genes, 4.7% | 137 out of 17817 genes, 0.8% | 8.59e-07 |
| Biological Process | hexose metabolic process | 33 out of 381 genes, 8.7% | 461 out of 17817 genes, 2.6% | 1.49e-06 |
| Biological Process | cellular response to stress | 40 out of 381 genes, 10.5% | 644 out of 17817 genes, 3.6% | 1.64e-06 |
| Biological Process | response to stress | 85 out of 381 genes, 22.3% | 2072 out of 17817 genes, 11.6% | 1.91e-06 |
| Biological Process | interphase | 22 out of 381 genes, 5.8% | 227 out of 17817 genes, 1.3% | 4.11e-06 |
| Biological Process | cellular nitrogen compound metabolic process | 123 out of 381 genes, 32.3% | 3532 out of 17817 genes, 19.8% | 4.69e-06 |
| Biological Process | organic cyclic compound metabolic process | 124 out of 381 genes, 32.5% | 3656 out of 17817 genes, 20.5% | 1.98e-05 |
| Biological Process | regulation of cell cycle | 32 out of 381 genes, 8.4% | 508 out of 17817 genes, 2.9% | 5.64e-05 |
| Biological Process | single-organism carbohydrate metabolic process | 35 out of 381 genes, 9.2% | 608 out of 17817 genes, 3.4% | 0.00012 |
| Biological Process | cellular glucan metabolic process | 10 out of 381 genes, 2.6% | 53 out of 17817 genes, 0.3% | 0.00016 |
| Biological Process | glucan metabolic process | 10 out of 381 genes, 2.6% | 53 out of 17817 genes, 0.3% | 0.00016 |
| Biological Process | protein catabolic process | 26 out of 381 genes, 6.8% | 379 out of 17817 genes, 2.1% | 0.00020 |
| Biological Process | polysaccharide metabolic process | 10 out of 381 genes, 2.6% | 57 out of 17817 genes, 0.3% | 0.00033 |
| Biological Process | cellular polysaccharide metabolic process | 10 out of 381 genes, 2.6% | 57 out of 17817 genes, 0.3% | 0.00033 |
| Biological Process | regulation of molecular function | 64 out of 381 genes, 16.8% | 1567 out of 17817 genes, 8.8% | 0.00035 |
| Biological Process | proteolysis | 28 out of 381 genes, 7.3% | 456 out of 17817 genes, 2.6% | 0.00066 |
| Biological Process | organophosphate metabolic process | 49 out of 381 genes, 12.9% | 1091 out of 17817 genes, 6.1% | 0.00071 |
| Biological Process | cellular aromatic compound metabolic process | 113 out of 381 genes, 29.7% | 3457 out of 17817 genes, 19.4% | 0.00084 |
| Biological Process | cellular carbohydrate metabolic process | 13 out of 381 genes, 3.4% | 112 out of 17817 genes, 0.6% | 0.00085 |
| Biological Process | proteolysis involved in cellular protein catabolic process | 24 out of 381 genes, 6.3% | 366 out of 17817 genes, 2.1% | 0.00136 |
| Biological Process | cellular protein catabolic process | 24 out of 381 genes, 6.3% | 367 out of 17817 genes, 2.1% | 0.00142 |
| Biological Process | carboxylic acid catabolic process | 13 out of 381 genes, 3.4% | 119 out of 17817 genes, 0.7% | 0.00170 |
| Biological Process | modification-dependent protein catabolic process | 23 out of 381 genes, 6.0% | 345 out of 17817 genes, 1.9% | 0.00171 |
| Biological Process | modification-dependent macromolecule catabolic process | 23 out of 381 genes, 6.0% | 345 out of 17817 genes, 1.9% | 0.00171 |
| Biological Process | organic acid catabolic process | 13 out of 381 genes, 3.4% | 121 out of 17817 genes, 0.7% | 0.00206 |
| Biological Process | phospholipid metabolic process | 17 out of 381 genes, 4.5% | 204 out of 17817 genes, 1.1% | 0.00207 |
| Biological Process | NAD metabolic process | 5 out of 381 genes, 1.3% | 12 out of 17817 genes, 0.1% | 0.00324 |
| Biological Process | monocarboxylic acid catabolic process | 10 out of 381 genes, 2.6% | 74 out of 17817 genes, 0.4% | 0.00401 |
| Biological Process | small molecule catabolic process | 13 out of 381 genes, 3.4% | 130 out of 17817 genes, 0.7% | 0.00463 |
| Biological Process | single-organism catabolic process | 13 out of 381 genes, 3.4% | 130 out of 17817 genes, 0.7% | 0.00463 |
| Biological Process | organic hydroxy compound metabolic process | 20 out of 381 genes, 5.2% | 292 out of 17817 genes, 1.6% | 0.00550 |
| Biological Process | acyl-CoA metabolic process | 8 out of 381 genes, 2.1% | 46 out of 17817 genes, 0.3% | 0.00553 |
| Biological Process | thioester metabolic process | 8 out of 381 genes, 2.1% | 46 out of 17817 genes, 0.3% | 0.00553 |
| Biological Process | regulation of fatty acid metabolic process | 7 out of 381 genes, 1.8% | 34 out of 17817 genes, 0.2% | 0.00672 |
| Biological Process | heterocycle metabolic process | 108 out of 381 genes, 28.3% | 3415 out of 17817 genes, 19.2% | 0.00797 |
| Biological Process | cellular modified amino acid metabolic process | 11 out of 381 genes, 2.9% | 99 out of 17817 genes, 0.6% | 0.00920 |
| Biological Process | macromolecule catabolic process | 33 out of 381 genes, 8.7% | 680 out of 17817 genes, 3.8% | 0.01145 |
| Biological Process | regulation of cellular ketone metabolic process | 8 out of 381 genes, 2.1% | 51 out of 17817 genes, 0.3% | 0.01231 |
| Biological Process | negative regulation of cellular process | 56 out of 381 genes, 14.7% | 1456 out of 17817 genes, 8.2% | 0.01295 |
| Biological Process | cellular process | 324 out of 381 genes, 85.0% | 13596 out of 17817 genes, 76.3% | 0.01552 |
| Biological Process | alcohol metabolic process | 17 out of 381 genes, 4.5% | 243 out of 17817 genes, 1.4% | 0.02135 |
| Biological Process | membrane lipid metabolic process | 12 out of 381 genes, 3.1% | 129 out of 17817 genes, 0.7% | 0.02258 |
| Biological Process | regulation of metabolic process | 109 out of 381 genes, 28.6% | 3542 out of 17817 genes, 19.9% | 0.02448 |
| Biological Process | fatty acid catabolic process | 9 out of 381 genes, 2.4% | 73 out of 17817 genes, 0.4% | 0.02624 |
| Biological Process | response to DNA damage stimulus | 24 out of 381 genes, 6.3% | 437 out of 17817 genes, 2.5% | 0.02722 |
| Biological Process | triglyceride metabolic process | 8 out of 381 genes, 2.1% | 57 out of 17817 genes, 0.3% | 0.02857 |
| Biological Process | mitotic cell cycle | 26 out of 381 genes, 6.8% | 501 out of 17817 genes, 2.8% | 0.03314 |
| Biological Process | cellular lipid catabolic process | 9 out of 381 genes, 2.4% | 76 out of 17817 genes, 0.4% | 0.03640 |
| Biological Process | production of molecular mediator involved in inflammatory response | 5 out of 381 genes, 1.3% | 19 out of 17817 genes, 0.1% | 0.04204 |
| Biological Process | protein metabolic process | 127 out of 381 genes, 33.3% | 4340 out of 17817 genes, 24.4% | 0.04389 |
| Biological Process | small molecule biosynthetic process | 11 out of 381 genes, 2.9% | 117 out of 17817 genes, 0.7% | 0.04499 |
| Biological Process | single-organism biosynthetic process | 11 out of 381 genes, 2.9% | 117 out of 17817 genes, 0.7% | 0.04499 |
| Cellular Component | intracellular part | 347 out of 360 genes, 96.4% | 13253 out of 17912 genes, 74.0% | 1.22e-28 |
| Cellular Component | intracellular | 347 out of 360 genes, 96.4% | 13314 out of 17912 genes, 74.3% | 5.19e-28 |
| Cellular Component | cell | 353 out of 360 genes, 98.1% | 14428 out of 17912 genes, 80.5% | 7.37e-23 |
| Cellular Component | cell part | 353 out of 360 genes, 98.1% | 14428 out of 17912 genes, 80.5% | 7.37e-23 |
| Cellular Component | mitochondrial part | 60 out of 360 genes, 16.7% | 654 out of 17912 genes, 3.7% | 3.30e-21 |
| Cellular Component | mitochondrion | 60 out of 360 genes, 16.7% | 660 out of 17912 genes, 3.7% | 5.29e-21 |
| Cellular Component | intracellular membrane-bounded organelle | 283 out of 360 genes, 78.6% | 9854 out of 17912 genes, 55.0% | 4.26e-19 |
| Cellular Component | membrane-bounded organelle | 283 out of 360 genes, 78.6% | 9953 out of 17912 genes, 55.6% | 2.91e-18 |
| Cellular Component | cytoplasmic part | 190 out of 360 genes, 52.8% | 5962 out of 17912 genes, 33.3% | 1.54e-12 |
| Cellular Component | cytoplasm | 190 out of 360 genes, 52.8% | 5986 out of 17912 genes, 33.4% | 2.39e-12 |
| Cellular Component | organelle envelope | 55 out of 360 genes, 15.3% | 885 out of 17912 genes, 4.9% | 8.78e-12 |
| Cellular Component | envelope | 55 out of 360 genes, 15.3% | 895 out of 17912 genes, 5.0% | 1.39e-11 |
| Cellular Component | intracellular organelle | 290 out of 360 genes, 80.6% | 11325 out of 17912 genes, 63.2% | 5.28e-11 |
| Cellular Component | organelle | 290 out of 360 genes, 80.6% | 11431 out of 17912 genes, 63.8% | 2.65e-10 |
| Cellular Component | organelle part | 184 out of 360 genes, 51.1% | 6284 out of 17912 genes, 35.1% | 2.94e-08 |
| Cellular Component | proteasome complex | 12 out of 360 genes, 3.3% | 54 out of 17912 genes, 0.3% | 7.55e-08 |
| Cellular Component | organelle membrane | 74 out of 360 genes, 20.6% | 1767 out of 17912 genes, 9.9% | 8.75e-08 |
| Cellular Component | intracellular organelle part | 167 out of 360 genes, 46.4% | 5689 out of 17912 genes, 31.8% | 4.49e-07 |
| Cellular Component | organelle inner membrane | 31 out of 360 genes, 8.6% | 519 out of 17912 genes, 2.9% | 9.15e-06 |
| Cellular Component | mitochondrial envelope | 26 out of 360 genes, 7.2% | 427 out of 17912 genes, 2.4% | 7.50e-05 |
| Cellular Component | mitochondrial tricarboxylic acid cycle enzyme complex | 4 out of 360 genes, 1.1% | 6 out of 17912 genes, 0.0% | 0.00030 |
| Cellular Component | tricarboxylic acid cycle enzyme complex | 4 out of 360 genes, 1.1% | 10 out of 17912 genes, 0.1% | 0.00398 |
| Cellular Component | proteasome accessory complex | 5 out of 360 genes, 1.4% | 21 out of 17912 genes, 0.1% | 0.00647 |
| Cellular Component | mitochondrial matrix | 8 out of 360 genes, 2.2% | 68 out of 17912 genes, 0.4% | 0.00827 |
| Cellular Component | protein complex | 75 out of 360 genes, 20.8% | 2447 out of 17912 genes, 13.7% | 0.01326 |
| Cellular Component | proteasome core complex | 4 out of 360 genes, 1.1% | 15 out of 17912 genes, 0.1% | 0.02389 |
| Molecular Function | catalytic activity | 287 out of 377 genes, 76.1% | 6353 out of 17883 genes, 35.5% | 5.26e-57 |
| Molecular Function | oxidoreductase activity | 68 out of 377 genes, 18.0% | 764 out of 17883 genes, 4.3% | 4.88e-22 |
| Molecular Function | cofactor binding | 27 out of 377 genes, 7.2% | 223 out of 17883 genes, 1.2% | 5.71e-11 |
| Molecular Function | transferase activity | 97 out of 377 genes, 25.7% | 2236 out of 17883 genes, 12.5% | 3.42e-10 |
| Molecular Function | hydrolase activity | 103 out of 377 genes, 27.3% | 2528 out of 17883 genes, 14.1% | 2.56e-09 |
| Molecular Function | hydrolase activity, acting on ester bonds | 44 out of 377 genes, 11.7% | 772 out of 17883 genes, 4.3% | 5.02e-07 |
| Molecular Function | coenzyme binding | 16 out of 377 genes, 4.2% | 114 out of 17883 genes, 0.6% | 5.37e-07 |
| Molecular Function | phosphatase activity | 24 out of 377 genes, 6.4% | 284 out of 17883 genes, 1.6% | 2.14e-06 |
| Molecular Function | peptidase activity | 38 out of 377 genes, 10.1% | 667 out of 17883 genes, 3.7% | 6.95e-06 |
| Molecular Function | peptidase activity, acting on L-amino acid peptides | 37 out of 377 genes, 9.8% | 656 out of 17883 genes, 3.7% | 1.38e-05 |
| Molecular Function | phosphoric ester hydrolase activity | 26 out of 377 genes, 6.9% | 363 out of 17883 genes, 2.0% | 1.52e-05 |
| Molecular Function | oxidoreductase activity, acting on the CH-CH group of donors | 11 out of 377 genes, 2.9% | 64 out of 17883 genes, 0.4% | 2.12e-05 |
| Molecular Function | transferase activity, transferring phosphorus-containing groups | 50 out of 377 genes, 13.3% | 1140 out of 17883 genes, 6.4% | 0.00016 |
| Molecular Function | small molecule binding | 87 out of 377 genes, 23.1% | 2487 out of 17883 genes, 13.9% | 0.00021 |
| Molecular Function | adenyl ribonucleotide binding | 63 out of 377 genes, 16.7% | 1602 out of 17883 genes, 9.0% | 0.00022 |
| Molecular Function | CoA-ligase activity | 5 out of 377 genes, 1.3% | 10 out of 17883 genes, 0.1% | 0.00023 |
| Molecular Function | oxidoreductase activity, acting on paired donors, with incorporation or reduction of molecular oxygen | 16 out of 377 genes, 4.2% | 177 out of 17883 genes, 1.0% | 0.00027 |
| Molecular Function | adenyl nucleotide binding | 63 out of 377 genes, 16.7% | 1614 out of 17883 genes, 9.0% | 0.00029 |
| Molecular Function | nucleotide binding | 82 out of 377 genes, 21.8% | 2360 out of 17883 genes, 13.2% | 0.00063 |
| Molecular Function | nucleoside phosphate binding | 82 out of 377 genes, 21.8% | 2360 out of 17883 genes, 13.2% | 0.00063 |
| Molecular Function | antioxidant activity | 9 out of 377 genes, 2.4% | 57 out of 17883 genes, 0.3% | 0.00067 |
| Molecular Function | isocitrate dehydrogenase activity | 4 out of 377 genes, 1.1% | 6 out of 17883 genes, 0.0% | 0.00069 |
| Molecular Function | oxidoreductase activity, acting on a sulfur group of donors | 9 out of 377 genes, 2.4% | 59 out of 17883 genes, 0.3% | 0.00091 |
| Molecular Function | ion binding | 118 out of 377 genes, 31.3% | 3841 out of 17883 genes, 21.5% | 0.00112 |
| Molecular Function | acyl-CoA dehydrogenase activity | 5 out of 377 genes, 1.3% | 14 out of 17883 genes, 0.1% | 0.00170 |
| Molecular Function | O-acyltransferase activity | 7 out of 377 genes, 1.9% | 36 out of 17883 genes, 0.2% | 0.00212 |
| Molecular Function | oxidoreductase activity, acting on CH-OH group of donors | 14 out of 377 genes, 3.7% | 163 out of 17883 genes, 0.9% | 0.00233 |
| Molecular Function | kinase activity | 35 out of 377 genes, 9.3% | 790 out of 17883 genes, 4.4% | 0.00721 |
| Molecular Function | succinate-CoA ligase activity | 3 out of 377 genes, 0.8% | 4 out of 17883 genes, 0.0% | 0.00900 |
| Molecular Function | endopeptidase activity | 24 out of 377 genes, 6.4% | 456 out of 17883 genes, 2.5% | 0.00991 |
| Molecular Function | transferase activity, transferring acyl groups | 16 out of 377 genes, 4.2% | 237 out of 17883 genes, 1.3% | 0.01120 |
| Molecular Function | cation binding | 110 out of 377 genes, 29.2% | 3710 out of 17883 genes, 20.7% | 0.01366 |
| Molecular Function | nucleoside kinase activity | 4 out of 377 genes, 1.1% | 12 out of 17883 genes, 0.1% | 0.02070 |
| Molecular Function | malate dehydrogenase activity | 3 out of 377 genes, 0.8% | 5 out of 17883 genes, 0.0% | 0.02215 |
| Molecular Function | peroxidase activity | 5 out of 377 genes, 1.3% | 25 out of 17883 genes, 0.1% | 0.03742 |
| Molecular Function | oxidoreductase activity, acting on peroxide as acceptor | 5 out of 377 genes, 1.3% | 25 out of 17883 genes, 0.1% | 0.03742 |
| Molecular Function | acylglycerol O-acyltransferase activity | 4 out of 377 genes, 1.1% | 14 out of 17883 genes, 0.1% | 0.04048 |
| Molecular function | phosphoprotein phosphatase activity | 12 out of 377 genes, 3.2% | 164 out of 17883 genes, 0.9% | 0.04704 |

TABLE 5: Significantly enriched pathways for DEGs from Con vs A/R (*P*<0.01).

| # | Pathway ^a^ | DEGs with pathway annotation (335) | All genes with pathway annotation (19680) | P value ^b^ | Q value ^c^ | Pathway ID |
| --- | --- | --- | --- | --- | --- | --- |
| 1 | Metabolic pathways | 81(24.18%) | 2021(10.27%) | 1.22E-13 | 2.57E-11 | ko01100 |
| 2 | Proteasome | 14(4.18%) | 72(0.37%) | 1.61E-11 | 1.69E-09 | ko03050 |
| 3 | Valine, leucine and isoleucine degradation | 12(3.58%) | 80(0.41%) | 1.04E-08 | 7.27E-07 | ko00280 |
| 4 | Pyruvate metabolism | 12(3.58%) | 93(0.47%) | 5.90E-08 | 3.10E-06 | ko00620 |
| 5 | Insulin signaling pathway | 18(5.37%) | 256(1.30%) | 4.42E-07 | 1.65E-05 | ko04910 |
| 6 | PPAR signaling pathway | 14(4.18%) | 156(0.79%) | 4.72E-07 | 1.65E-05 | ko03320 |
| 7 | Propanoate metabolism | 9(2.69%) | 60(0.30%) | 7.43E-07 | 2.23E-05 | ko00640 |
| 8 | Peroxisome | 13(3.88%) | 152(0.77%) | 2.09E-06 | 5.49E-05 | ko04146 |
| 9 | Citrate cycle (TCA cycle) | 7(2.09%) | 40(0.20%) | 4.47E-06 | 1.04E-04 | ko00020 |
| 10 | alpha-Linolenic acid metabolism | 7(2.09%) | 43(0.22%) | 7.40E-06 | 1.55E-04 | ko00592 |
| 11 | Biosynthesis of unsaturated fatty acids | 7(2.09%) | 45(0.23%) | 1.01E-05 | 1.93E-04 | ko01040 |
| 12 | Glyoxylate and dicarboxylate metabolism | 6(1.79%) | 34(0.17%) | 2.09E-05 | 3.66E-04 | ko00630 |
| 13 | Fatty acid metabolism | 8(2.39%) | 75(0.38%) | 4.06E-05 | 6.56E-04 | ko00071 |
| 14 | Synthesis and degradation of ketone bodies | 4(1.19%) | 13(0.07%) | 5.22E-05 | 7.37E-04 | ko00072 |
| 15 | Arginine and proline metabolism | 9(2.69%) | 100(0.51%) | 5.27E-05 | 7.37E-04 | ko00330 |
| 16 | beta-Alanine metabolism | 6(1.79%) | 46(0.23%) | 0.000123 | 1.61E-03 | ko00410 |
| 17 | Pentose and glucuronate interconversions | 6(1.79%) | 49(0.25%) | 0.000176 | 2.17E-03 | ko00040 |
| 18 | Ascorbate and aldarate metabolism | 5(1.49%) | 32(0.16%) | 0.000191 | 2.23E-03 | ko00053 |
| 19 | Butanoate metabolism | 6(1.79%) | 52(0.26%) | 0.000245 | 2.71E-03 | ko00650 |
| 20 | Long-term potentiation | 9(2.69%) | 132(0.67%) | 0.000437 | 4.59E-03 | ko04720 |
| 21 | Histidine metabolism | 5(1.49%) | 39(0.20%) | 0.000497 | 4.97E-03 | ko00340 |
| 22 | Primary bile acid biosynthesis | 5(1.49%) | 40(0.20%) | 0.00056 | 5.34E-03 | ko00120 |
| 23 | Tryptophan metabolism | 6(1.79%) | 65(0.33%) | 0.000826 | 7.54E-03 | ko00380 |
| 24 | Pentose phosphate pathway | 5(1.49%) | 47(0.24%) | 0.001184 | 1.04E-02 | ko00030 |
| 25 | Glycolysis / Gluconeogenesis | 14(4.18%) | 322(1.64%) | 0.001319 | 1.11E-02 | ko00010 |
| 26 | Glycerolipid metabolism | 7(2.09%) | 98(0.50%) | 0.001432 | 1.12E-02 | ko00561 |
| 27 | Protein processing in endoplasmic reticulum | 13(3.88%) | 289(1.47%) | 0.001436 | 1.12E-02 | ko04141 |
| 28 | Amino sugar and nucleotide sugar metabolism | 6(1.79%) | 74(0.38%) | 0.00163 | 1.22E-02 | ko00520 |
| 29 | Ubiquinone and other terpenoid-quinone biosynthesis | 3(0.90%) | 15(0.08%) | 0.001911 | 1.38E-02 | ko00130 |
| 30 | Fructose and mannose metabolism | 6(1.79%) | 88(0.45%) | 0.003914 | 2.74E-02 | ko00051 |
| 31 | VEGF signaling pathway | 8(2.39%) | 152(0.77%) | 0.004575 | 3.10E-02 | ko04370 |
| 32 | Oocyte meiosis | 10(2.99%) | 221(1.12%) | 0.004734 | 3.11E-02 | ko04114 |
| 33 | SNARE interactions in vesicular transport | 4(1.19%) | 42(0.21%) | 0.005547 | 3.36E-02 | ko04130 |
| 34 | Renal cell carcinoma | 7(2.09%) | 125(0.64%) | 0.005603 | 3.36E-02 | ko05211 |
| 35 | Adipocytokine signaling pathway | 7(2.09%) | 125(0.64%) | 0.005603 | 3.36E-02 | ko04920 |
| 36 | Inositol phosphate metabolism | 6(1.79%) | 96(0.49%) | 0.005983 | 3.49E-02 | ko00562 |
| 37 | Nicotinate and nicotinamide metabolism | 4(1.19%) | 45(0.23%) | 0.007096 | 4.03E-02 | ko00760 |
| 38 | Lysosome | 9(2.69%) | 200(1.02%) | 0.007457 | 4.12E-02 | ko04142 |
| 39 | mTOR signaling pathway | 6(1.79%) | 104(0.53%) | 0.008754 | 4.71E-02 | ko04150 |
| 40 | Amyotrophic lateral sclerosis (ALS) | 7(2.09%) | 138(0.70%) | 0.009457 | 4.96E-02 | ko05014 |
|  |  |  |  |  |  |  |

^a^ Pathway analysis based on KOBAS server 2.0[1, 2];

^b^ P-value in hypergeometric test;

^c^ The Q-value is similar to the well known *p*-value, except it is a measure of significance in terms of the false discovery rate rather than the false positive rate[3].

TABLE 6: Significantly enriched pathways for DEGs from A/R vs DZ (*P*<0.01).

| # | Pathway ^a^ | DEGs with pathway annotation (467) | All genes with pathway annotation (19680) | P value ^b^ | Q value ^c^ | Pathway ID |
| --- | --- | --- | --- | --- | --- | --- |
| 1 | Metabolic pathways | 143(30.62%) | 2021(10.27%) | 9.09E-35 | 1.97E-32 | ko01100 |
| 2 | Proteasome | 20(4.28%) | 72(0.37%) | 2.14E-16 | 2.32E-14 | ko03050 |
| 3 | Valine, leucine and isoleucine degradation | 19(4.07%) | 80(0.41%) | 2.87E-14 | 2.07E-12 | ko00280 |
| 4 | Citrate cycle (TCA cycle) | 14(3.00%) | 40(0.20%) | 1.95E-13 | 1.06E-11 | ko00020 |
| 5 | Pyruvate metabolism | 16(3.43%) | 93(0.47%) | 5.58E-10 | 2.42E-08 | ko00620 |
| 6 | Propanoate metabolism | 13(2.78%) | 60(0.30%) | 1.20E-09 | 4.34E-08 | ko00640 |
| 7 | Fatty acid metabolism | 14(3.00%) | 75(0.38%) | 2.21E-09 | 6.85E-08 | ko00071 |
| 8 | Glycerolipid metabolism | 14(3.00%) | 98(0.50%) | 7.82E-08 | 2.12E-06 | ko00561 |
| 9 | Arginine and proline metabolism | 14(3.00%) | 100(0.51%) | 1.01E-07 | 2.45E-06 | ko00330 |
| 10 | Peroxisome | 17(3.64%) | 152(0.77%) | 1.30E-07 | 2.82E-06 | ko04146 |
| 11 | Insulin signaling pathway | 22(4.71%) | 256(1.30%) | 2.22E-07 | 4.38E-06 | ko04910 |
| 12 | Glycerophospholipid metabolism | 15(3.21%) | 134(0.68%) | 7.03E-07 | 1.27E-05 | ko00564 |
| 13 | Glutathione metabolism | 12(2.57%) | 87(0.44%) | 9.80E-07 | 1.45E-05 | ko00480 |
| 14 | PPAR signaling pathway | 16(3.43%) | 156(0.79%) | 9.88E-07 | 1.45E-05 | ko03320 |
| 15 | Glyoxylate and dicarboxylate metabolism | 8(1.71%) | 34(0.17%) | 1.00E-06 | 1.45E-05 | ko00630 |
| 16 | beta-Alanine metabolism | 8(1.71%) | 46(0.23%) | 1.12E-05 | 1.52E-04 | ko00410 |
| 17 | Tryptophan metabolism | 9(1.93%) | 65(0.33%) | 2.17E-05 | 2.77E-04 | ko00380 |
| 18 | Galactose metabolism | 8(1.71%) | 53(0.27%) | 3.29E-05 | 3.96E-04 | ko00052 |
| 19 | Oocyte meiosis | 16(3.43%) | 221(1.12%) | 8.10E-05 | 9.14E-04 | ko04114 |
| 20 | Biosynthesis of unsaturated fatty acids | 7(1.50%) | 45(0.23%) | 8.42E-05 | 9.14E-04 | ko01040 |
| 21 | Ascorbate and aldarate metabolism | 6(1.28%) | 32(0.16%) | 9.28E-05 | 9.58E-04 | ko00053 |
| 22 | Synthesis and degradation of ketone bodies | 4(0.86%) | 13(0.07%) | 0.000189 | 1.76E-03 | ko00072 |
| 23 | Renal cell carcinoma | 11(2.36%) | 125(0.64%) | 0.000195 | 1.76E-03 | ko05211 |
| 24 | Adipocytokine signaling pathway | 11(2.36%) | 125(0.64%) | 0.000195 | 1.76E-03 | ko04920 |
| 25 | Fructose and mannose metabolism | 9(1.93%) | 88(0.45%) | 0.00024 | 2.08E-03 | ko00051 |
| 26 | Glycolysis / Gluconeogenesis | 19(4.07%) | 322(1.64%) | 0.00027 | 2.26E-03 | ko00010 |
| 27 | Histidine metabolism | 6(1.28%) | 39(0.20%) | 0.00029 | 2.33E-03 | ko00340 |
| 28 | Long-term potentiation | 11(2.36%) | 132(0.67%) | 0.000314 | 2.43E-03 | ko04720 |
| 29 | Amino sugar and nucleotide sugar metabolism | 8(1.71%) | 74(0.38%) | 0.000362 | 2.71E-03 | ko00520 |
| 30 | alpha-Linolenic acid metabolism | 6(1.28%) | 43(0.22%) | 0.000501 | 3.62E-03 | ko00592 |
| 31 | Nicotinate and nicotinamide metabolism | 6(1.28%) | 45(0.23%) | 0.000643 | 4.50E-03 | ko00760 |
| 32 | Pentose phosphate pathway | 6(1.28%) | 47(0.24%) | 0.000814 | 5.44E-03 | ko00030 |
| 33 | mTOR signaling pathway | 9(1.93%) | 104(0.53%) | 0.000828 | 5.44E-03 | ko04150 |
| 34 | Pentose and glucuronate interconversions | 6(1.28%) | 49(0.25%) | 0.001019 | 6.32E-03 | ko00040 |
| 35 | Lysosome | 13(2.78%) | 200(1.02%) | 0.001019 | 6.32E-03 | ko04142 |
| 36 | Sphingolipid metabolism | 7(1.50%) | 69(0.35%) | 0.001229 | 7.41E-03 | ko00600 |
| 37 | Pantothenate and CoA biosynthesis | 4(0.86%) | 21(0.11%) | 0.001359 | 7.97E-03 | ko00770 |
| 38 | Butanoate metabolism | 6(1.28%) | 52(0.26%) | 0.001397 | 7.98E-03 | ko00650 |
| 39 | Bladder cancer | 7(1.50%) | 75(0.38%) | 0.002003 | 1.11E-02 | ko05219 |
| 40 | Cysteine and methionine metabolism | 7(1.50%) | 76(0.39%) | 0.002162 | 1.17E-02 | ko00270 |
| 41 | Primary bile acid biosynthesis | 5(1.07%) | 40(0.20%) | 0.002444 | 1.29E-02 | ko00120 |
| 42 | Alanine, aspartate and glutamate metabolism | 5(1.07%) | 44(0.22%) | 0.003733 | 1.93E-02 | ko00250 |
| 43 | Protein processing in endoplasmic reticulum | 15(3.21%) | 289(1.47%) | 0.00398 | 2.01E-02 | ko04141 |
| 44 | Starch and sucrose metabolism | 6(1.28%) | 65(0.33%) | 0.004381 | 2.16E-02 | ko00500 |
| 45 | Ubiquinone and other terpenoid-quinone biosynthesis | 3(0.64%) | 15(0.08%) | 0.004884 | 2.36E-02 | ko00130 |
| 46 | Tyrosine metabolism | 6(1.28%) | 68(0.35%) | 0.005471 | 2.58E-02 | ko00350 |
| 47 | Neurotrophin signaling pathway | 13(2.78%) | 254(1.29%) | 0.007918 | 3.66E-02 | ko04722 |
| 48 | Chronic myeloid leukemia | 9(1.93%) | 148(0.75%) | 0.008908 | 4.03E-02 | ko05220 |

^a^ Pathway analysis based on KOBAS server 2.0[1, 2];

^b^ P-value in hypergeometric test;

^c^ The Q-value is similar to the well known *p*-value, except it is a measure of significance in terms of the false discovery rate rather than the false positive rate[3].

TABLE 7: Significantly enriched pathways for DEGs from DZ vs DZ5HD (*P*<0.01).

| # | Pathway ^a^ | DEGs with pathway annotation (361) | All genes with pathway annotation (19680) | P value ^b^ | Q value ^c^ | Pathway ID |
| --- | --- | --- | --- | --- | --- | --- |
| 1 | Metabolic pathways | 106(29.36%) | 2021(10.27%) | 2.26E-24 | 4.85E-22 | ko01100 |
| 2 | Proteasome | 16(4.43%) | 72(0.37%) | 1.91E-13 | 2.06E-11 | ko03050 |
| 3 | Citrate cycle (TCA cycle) | 12(3.32%) | 40(0.20%) | 4.26E-12 | 3.05E-10 | ko00020 |
| 4 | Valine, leucine and isoleucine degradation | 12(3.32%) | 80(0.41%) | 2.38E-08 | 1.28E-06 | ko00280 |
| 5 | Propanoate metabolism | 10(2.77%) | 60(0.30%) | 1.27E-07 | 5.47E-06 | ko00640 |
| 6 | PPAR signaling pathway | 14(3.88%) | 156(0.79%) | 1.15E-06 | 4.12E-05 | ko03320 |
| 7 | Biosynthesis of unsaturated fatty acids | 8(2.22%) | 45(0.23%) | 1.41E-06 | 4.34E-05 | ko01040 |
| 8 | Glutathione metabolism | 10(2.77%) | 87(0.44%) | 4.35E-06 | 1.17E-04 | ko00480 |
| 9 | Fatty acid metabolism | 9(2.49%) | 75(0.38%) | 9.21E-06 | 2.20E-04 | ko00071 |
| 10 | Arginine and proline metabolism | 10(2.77%) | 100(0.51%) | 1.52E-05 | 3.28E-04 | ko00330 |
| 11 | Peroxisome | 12(3.32%) | 152(0.77%) | 2.46E-05 | 4.82E-04 | ko04146 |
| 12 | Glycerophospholipid metabolism | 11(3.05%) | 134(0.68%) | 3.74E-05 | 6.69E-04 | ko00564 |
| 13 | Oocyte meiosis | 14(3.88%) | 221(1.12%) | 6.19E-05 | 1.02E-03 | ko04114 |
| 14 | Glycerolipid metabolism | 9(2.49%) | 98(0.50%) | 7.97E-05 | 1.22E-03 | ko00561 |
| 15 | alpha-Linolenic acid metabolism | 6(1.66%) | 43(0.22%) | 0.000126 | 1.80E-03 | ko00592 |
| 16 | Pyruvate metabolism | 8(2.22%) | 93(0.47%) | 0.000311 | 4.18E-03 | ko00620 |
| 17 | Pantothenate and CoA biosynthesis | 4(1.11%) | 21(0.11%) | 0.000521 | 6.58E-03 | ko00770 |
| 18 | Long-term potentiation | 9(2.49%) | 132(0.67%) | 0.000746 | 8.91E-03 | ko04720 |
| 19 | Tryptophan metabolism | 6(1.66%) | 65(0.33%) | 0.001214 | 1.37E-02 | ko00380 |
| 20 | Synthesis and degradation of ketone bodies | 3(0.83%) | 13(0.07%) | 0.001527 | 1.64E-02 | ko00072 |
| 21 | Sphingolipid metabolism | 6(1.66%) | 69(0.35%) | 0.001657 | 1.70E-02 | ko00600 |
| 22 | p53 signaling pathway | 9(2.49%) | 151(0.77%) | 0.001918 | 1.87E-02 | ko04115 |
| 23 | Bladder cancer | 6(1.66%) | 75(0.38%) | 0.00254 | 2.37E-02 | ko05219 |
| 24 | Cysteine and methionine metabolism | 6(1.66%) | 76(0.39%) | 0.002716 | 2.37E-02 | ko00270 |
| 25 | Protein processing in endoplasmic reticulum | 13(3.60%) | 289(1.47%) | 0.002759 | 2.37E-02 | ko04141 |
| 26 | Glyoxylate and dicarboxylate metabolism | 4(1.11%) | 34(0.17%) | 0.003344 | 2.77E-02 | ko00630 |
| 27 | Fatty acid elongation | 4(1.11%) | 38(0.19%) | 0.005026 | 4.00E-02 | ko00062 |
| 28 | Dopaminergic synapse | 10(2.77%) | 210(1.07%) | 0.005562 | 4.16E-02 | ko04728 |
| 29 | Fructose and mannose metabolism | 6(1.66%) | 88(0.45%) | 0.00561 | 4.16E-02 | ko00051 |
| 30 | Primary bile acid biosynthesis | 4(1.11%) | 40(0.20%) | 0.006047 | 4.33E-02 | ko00120 |
| 31 | Phenylalanine metabolism | 4(1.11%) | 41(0.21%) | 0.006605 | 4.58E-02 | ko00360 |
| 32 | Insulin signaling pathway | 11(3.05%) | 256(1.30%) | 0.007912 | 5.25E-02 | ko04910 |
| 33 | Tyrosine metabolism | 5(1.39%) | 68(0.35%) | 0.008192 | 5.25E-02 | ko00350 |
| 34 | Renal cell carcinoma | 7(1.94%) | 125(0.64%) | 0.008305 | 5.25E-02 | ko05211 |
| 35 | MAPK signaling pathway | 16(4.43%) | 449(2.28%) | 0.009117 | 5.45E-02 | ko04010 |
| 36 | Cell cycle | 10(2.77%) | 226(1.15%) | 0.00913 | 5.45E-02 | ko04110 |
| 37 | beta-Alanine metabolism | 4(1.11%) | 46(0.23%) | 0.009911 | 5.76E-02 | ko00410 |

^a^ Pathway analysis based on KOBAS server 2.0[1, 2];

^b^ P-value in hypergeometric test;

^c^ The Q-value is similar to the well known *p*-value, except it is a measure of significance in terms of the false discovery rate rather than the false positive rate[3].

**References**

[1] C. Xie, X. Mao, J. Huang, Y. Ding, J. Wu, S. Dong, L. Kong, G. Gao, C.-Y. Li, and L. Wei, "KOBAS 2.0: a web server for annotation and identification of enriched pathways and diseases," *Nucleic acids research*, vol. 39, no. suppl 2, pp. W316-W322, 2011.

[2] J. Wu, X. Mao, T. Cai, J. Luo, and L. Wei, "KOBAS server: a web-based platform for automated annotation and pathway identification," *Nucleic acids research*, vol. 34, no. suppl 2, pp. W720-W724, 2006.

[3] J. D. Storey, and R. Tibshirani, "Statistical significance for genomewide studies," P*roceedings of the National Academy of Sciences*, vol. 100, no. 16, pp. 9440-9445, 2003.
